# Monetary incentives for improving smartphone-measured oral hygiene behaviors in young children: A randomized pilot trial

**DOI:** 10.1371/journal.pone.0236692

**Published:** 2020-07-30

**Authors:** Justin S. White, Francisco Ramos-Gomez, Jenny X. Liu, Bonnie Jue, Tracy L. Finlayson, Jeremiah R. Garza, Alexandra H. Crawford, Sarit Helman, William Santo, Jing Cheng, James G. Kahn, Stuart A. Gansky

**Affiliations:** 1 Philip R. Lee Institute for Health Policy Studies, University of California San Francisco, San Francisco, California, United States of America; 2 Center to Address Disparities in Children’s Oral Health, University of California San Francisco, San Francisco, California, United States of America; 3 Section of Pediatric Dentistry, School of Dentistry, University of California Los Angeles, Los Angeles, California, United States of America; 4 Institute for Health and Aging, University of California San Francisco, San Francisco, California, United States of America; 5 Department of Preventive and Restorative Dental Sciences, University of California San Francisco, San Francisco, California, United States of America; 6 School of Public Health, San Diego State University, San Diego, California, United States of America; 7 California Protons Cancer Therapy Center, University of California San Diego Health, San Diego, California, United States of America; 8 Bakar Computational Health Sciences Institute, University of California San Francisco, San Francisco, California, United States of America; The University of Manchester Faculty of Biology Medicine and Health, UNITED KINGDOM

## Abstract

**Aims:**

To assess feasibility, acceptability, and early efficacy of monetary incentive-based interventions on fostering oral hygiene in young children measured with a Bluetooth-enabled toothbrush and smartphone application.

**Design:**

A stratified, parallel-group, three-arm individually randomized controlled pilot trial.

**Setting:**

Two Los Angeles area Early Head Start (EHS) sites.

**Participants:**

36 parent-child dyads enrolled in an EHS home visit program for 0–3 year olds.

**Interventions:**

Eligible dyads, within strata and permuted blocks, were randomized in equal allocation to one of three groups: waitlist (delayed monetary incentive) control group, fixed monetary incentive package, or lottery monetary incentive package. The intervention lasted 8 weeks.

**Outcomes:**

Primary outcomes were a) toothbrushing performance: mean number of Bluetooth-recorded half-day episodes per week when the child’s teeth were brushed, and b) dental visit by the 2-month follow-up among children with no prior dental visit. The *a priori* milestone of 20% more frequent toothbrushing identified the intervention for a subsequent trial. Feasibility and acceptability measures were also assessed, including frequency of parents syncing the Bluetooth-enabled toothbrush to the smartphone application and plaque measurement from digital photographs.

**Findings:**

Digital monitoring of toothbrushing was feasible. Mean number of weekly toothbrushing episodes over 8 weeks was 3.9 in the control group, 4.1 in the fixed incentive group, and 6.0 in the lottery incentive group. The lottery group had 53% more frequent toothbrushing than the control group and 47% more frequent toothbrushing than the fixed group. Exploratory analyses showed effects concentrated among children ≤24 months. Follow-up dental visit attendance was similar across groups. iPhone 7 more reliably captured evaluable images than Photomed Cannon G16.

**Conclusions:**

Trial protocol and outcome measures were deemed feasible and acceptable. Results informed the study protocol for a fully powered trial of lottery incentives versus a delayed control using the smart toothbrush and remote digital incentive program administration.

**Trial registration:**

ClinicalTrials.gov identifier NCT03862443.

## Introduction

Childhood caries remains the most prevalent chronic childhood disease in the United States [1]. Early childhood caries (ECC)—defined as ≥1 decayed, lost, or restored tooth surface by age 71 months—poses a serious threat to child welfare, particularly among economically disadvantaged, underserved, and migrant children [2, 3]. Initiating early prevention may contribute to forming and maintaining healthy dental habits, thereby preventing disease and sequelae [4].

Personal incentives are commonly part of health promotion programs but have not yet been used in dentistry. For example, over three-quarters of large US employers offer monetary incentives through wellness programs [5]; publicly funded programs often provide subsidies or vouchers for food, family planning services, and other preventive health products. Several studies have shown that incentives can effectively promote a wide range of preventive health behaviors [6, 7]. Despite evidence supporting the effectiveness of incentives and their popularity, their efficacy for encouraging oral health management remains untested.

Behavioral economics has established that incentive structure may partly determine effectiveness [8]. Lottery incentives are one “behavioral” incentive design showing promise. Lottery incentives leverage the tendency of individuals to be more motivated by immediate rather than delayed gratification [9], to over-react to low-probability events [10, 11], and to avoid feeling regret [12–15]. Most incentive programs use single-tiered lotteries in which participants have a fixed probability of winning a reward. If a program increases the reward amount, it typically offsets the added cost by decreasing the probability of winning, and infrequent reinforcement of the behavior can demotivate individuals. If a program increases the probability of winning, it typically offsets the added cost by decreasing the reward amount, and a smaller reward can be demotivating—known as the peanuts effect [16, 17].

Combined or 2-tiered lotteries try to improve on single-tier lotteries via a better balance between frequent reinforcement and large potential rewards. Specifically, combined lotteries offer both a higher probability of a smaller reward and a lower probability of a larger reward. Researchers have found combined lottery incentives promote weight loss [18], physical activity [19], medication adherence [20, 21], and home monitoring of glucose levels [22]. These programs have been administered remotely using widely available digital technologies: digital monitors or sensors to track behavior and text-messaging or smartphone applications (apps) to deliver feedback to users. Smartphone toothbrushing apps have shown promising efficacy in small trials [23, 24]. To our knowledge combined lottery programs have neither been tested for promoting oral health management in adults or children nor for other health behaviors (e.g., diet) where parents supervise or administer the behavior of young children. Moreover, only a handful of studies—all in low-income countries—have tested lotteries for health promotion among a low-income population [25–27].

The BEECON (Behavioral Economics for Oral Health Innovation) pilot trial was implemented in Early Head Start (EHS) sites in the Los Angeles, CA area. Our objective was to test the feasibility, acceptability, and short-term proof of concept of a fixed monetary incentive package and combined lottery incentive package compared to a waitlist (delayed incentive) control group in a pilot randomized controlled trial (RCT) to encourage low-income parents of children under age 4 to brush their children’s teeth regularly and take their children to regularly scheduled dental visits. The study aimed to generate hypotheses and provide preliminary data to identify an incentive-based intervention, calculate sample size, and inform recruitment and retention for a future full-scale trial. To track toothbrushing adherence and administer the incentives remotely, all parents used a Bluetooth-enabled toothbrush that synchronized data on brushing episodes to a smartphone app and subsequently received automated texts with feedback on toothbrushing performance and monetary incentives earned.

## Methods

### Trial design

We conducted a parallel-group, three-arm, stratified, open-label, pilot RCT equally allocating parent-child dyads to three intervention groups, specified below. The BEECON pilot project adopted a mixed-methods approach, including self-reported questionnaires, dental examinations, and qualitative interviews of study participants [28]. The study protocol is provided in the supporting online information with a CONSORT guideline checklist [29]. The UCSF institutional review board (IRB), as the reviewing IRB with the UCLA IRB as the relying IRB, reviewed and approved study procedures. The funder and its appointed External Scientific Committee reviewed study procedures before launch. Because the funder did not require completing official trial registration, the trial was retrospectively registered at ClinicalTrials.gov (NCT03862443). The authors confirm that all ongoing and related trials for this drug/intervention are registered. No changes to the pre-specified trial design and methods were made after trial commencement. During an initial clinic visit, all participating parents signed an informed consent form that included consent to photographing their child’s teeth and testing the Bluetooth-enabled powered toothbrush at home with their child. In-person study procedures occurred at baseline and at the 8-week follow-up visits.

### Recruitment and participants

Inclusion criteria were: be a parent or caregiver of a 6 to 42 month-old with at least 2 fully erupted teeth; aged 18 and older; speak, read, and write either English or Spanish; have a child enrolled or waitlisted in a participating Early Head Start (EHS) home visit program; not planning to move for the next 6 months outside the greater Los Angeles area; own a smartphone with Google Play or iTunes stores and be willing to download the free smartphone toothbrush app; be willing to be contacted via text-messaging for study-related notifications; provide informed consent in English or Spanish; and agree to comply with all study procedures and be available for the study duration.

Exclusion criteria for the children were: known allergic reaction to any study product component; being uncooperative or behaviorally unsuited during a screening visit toothbrushing prophylaxis; being a sibling of a study enrolled child (the family’s oldest eligible child was the study child); being enrolled in foster care; and anything else putting the child at increased risk or precluding full compliance with, or completing, the study.

The study recruited individuals enrolled in, or waitlisted at, the Venice Family Clinic’s Children First EHS home visit program in Santa Monica and Culver City, CA, near Los Angeles. Participants were recruited through outreach to families during EHS monthly parent meetings, and via telephone calls to interested parents/caregivers who contacted study staff. Study recruitment information was included in monthly EHS newsletters and flyers distributed at EHS centers and parent meetings. Enrollment occurred from May-June 2017. Potential participants were screened for eligibility using a structured questionnaire during a telephone interview with study personnel.

### Randomization and masking

Eligible consenting parent-child participant dyads, within strata and permuted blocks, were randomized with equal allocation to one of three groups: a control group, a fixed incentive group, or a lottery incentive group. An independent Consortium Coordinating Center statistician generated the randomization sequence using randomization stratified by parent’s smartphone operating system (iOS or Android), and permuted blocks of varying unknown sizes divisible by 3, concealing the sequence from study staff and investigators. Dyads were also randomized to the sequence of two cameras for imaging the secondary plaque measurement. The randomization schedule was securely stored in the Research Electronic Data Capture (REDCap, version 9.1.3, Nashville, TN), as the clinical trials management system hosted at the University of California, San Francisco using a custom-designed nested randomization implementation [30, 31]. REDCap is secure, web-based software supporting clinical research data capture which includes 1) a validated data capture interface, 2) audit trails for tracking data edits and exports, 3) automated export for download to popular statistical packages, and 4) data integration and external source interoperability tools. Independent dental examiners were blinded to participants’ group assignment; staff members instructed participants not to mention the incentive program during the dental exams.

### Procedures

An initial study visit was scheduled after parents had provided written informed consent to participate in the study. During the visit, the toothbrushing app was downloaded onto the participating parent’s smartphone, and study personnel demonstrated using the powered toothbrush and toothpaste pump and properly synchronizing the toothbrush to the parent’s smartphone; parents completed a baseline questionnaire. The baseline questionnaire assessed demographic and oral health characteristics and behaviors. The participating children underwent baseline assessments: dental screening, plaque disclosing solution application, extraoral photographs, fluoride varnish application, and standardized anticipatory guidance.

The toothbrushing intervention consisted of a “practice” week starting the day after baseline, and 8 study weeks (56 days) starting Monday after the (partial) practice week. A practice week allowed participants to familiarize themselves with the technology and procedure. During the study period, participants used the powered toothbrush, synchronized toothbrushing data to the app, and received SMS messages, as detailed below.

Participants were asked to return for a follow-up at 8 weeks, where participants repeated baseline assessments. During follow-up, parents provided self-reported daily toothbrushing diary data, and both incentive group participants received their cumulative earned incentives.

#### Study interventions

During the initial study visit, study personnel used visual presentations and teach-back techniques to convey information on the behavioral study interventions. A description of each intervention follows.

*Waitlist (delayed monetary incentive) control group*. In the control group, participants were ineligible for performance-based incentives during the 8 study weeks, but received information on their toothbrushing performance per the powered toothbrush data, as well as SMS brushing performance messages and reminders to synchronize toothbrushing data to the app. After the 8-week follow-up, the control group could then earn money identical to the fixed incentive group in an 8-week, open-label extension, which is not part of the formal evaluation, but rather a supplemental analysis. This delayed design provided all participating EHS parents the chance to earn the same monetary incentives.

*Fixed monetary incentive group*. In the fixed incentive group, participants were eligible to earn one of two incentive amounts every week: (1) $5 if the participant met a lower performance threshold (7 episodes, at least once per day, of brushing child’s teeth ≥1 minute) during the preceding week, and (2) $10 if the participant met a higher performance threshold (14 episodes, twice per day, ≥1 minute) during the preceding week. Participants in this group also received SMS brushing performance messages and reminders to synchronize toothbrushing data to the app.

*Lottery monetary incentive group*. In the lottery incentive group, participants were sent an SMS message about being entered into a lottery drawing every week; the participant could text back a 2-digit number from 00 to 99 or let her number be chosen at random. The computer-generated winning number was announced by SMS message on the Tuesday after each study week. The probability of winning depended on the participant’s performance level. We used two performance thresholds: a lower performance threshold of 7 episodes ≥1 minute per week (at least once per day) and a higher performance threshold of 14 episodes ≥1 minute per week (twice per day).

A participant who met the *lower* performance threshold won $25 if one digit matched the winning number in order (18% probability) and $50 if both digits matched in order (1% probability). A participant who met the *higher* performance threshold won $25 if one digit matched the winning number irrespective of order (34% probability) and $50 if both digits matched irrespective of order (4% probability) (S1 Table). Thus, the chance of winning roughly doubled for those meeting the higher vs. lower performance threshold. The total expected value was $5 per week for the lower performance lottery and $10 per week for the higher performance lottery, identical to the rewards in the fixed incentive group. The incentive amounts were selected to be comparable to prior studies that have used daily lottery incentives for health behavior maintenance [32, 33].

Participants who failed to reach either performance threshold were entered into the lower performance drawing, and if chosen as a winner received an SMS message stating what they would have won had they brushed more regularly, thereby taking advantage of a psychological tendency toward anticipated regret to motivate future brushing [15]. Participants in this group also received SMS brushing performance messages and reminders to synchronize toothbrushing data to the app.

All incentive payments were provided as redeemable gift cards at the 2-month visit.

#### Anticipatory guidance

During each dental assessment, parents were given an electronic tablet to watch a short (3 to 5 minute) anticipatory guidance health education video in English (https://youtu.be/qiabuZLkZ-A) or Spanish (https://youtu.be/bBWPjU1uCKM), developed by the UCLA study team. The video contained key preventive oral health messages appropriate for the child’s age, such as the importance of fluoride supplements, healthy snacking, and preventing transmission of bacteria that cause caries [34]. Research staff also read a script that recommended brushing with fluoridated toothpaste at least twice a day, once in the morning and once at night before bed, for either one minute (for children with 2–10 teeth) or 2 minutes (for children with >10 teeth) per episode.

#### Tracking toothbrushing performance and frequency

*Bluetooth-enabled powered toothbrush*. During the baseline visit, all participants received a Bluetooth-enabled powered toothbrush (Sonicare for Kids, Philips Healthcare, Andover, MA). The toothbrush used Bluetooth technology to passively transmit data on brushing timing, frequency, and duration from the toothbrush to the Sonicare for Kids (S4K) app installed on the participant’s smartphone. The S4K app is designed to enhance child engagement using an animated character named Sparkly and “gamification” techniques (e.g., badges earned for frequent toothbrushing), to track toothbrushing behavior, and to provide toothbrushing advice and healthy tips. Parents were instructed to synchronize (download) data from the powered toothbrush to their phone app every Wednesday, Friday, and Sunday of the study period. The app then synchronized the toothbrushing data to remote server storage (the cloud). Every week, the vendor transmitted participants’ brushing data from the remote server to study investigators. To increase completeness of brushing outcome data, parents in all study groups were paid $3 per study week in which they synchronized their toothbrush three times, regardless of the number of brushing episodes.

*Calendar diaries*. Parents were given paper calendar diaries to self-report toothbrushing events to compare to the powered toothbrush’s data. Research staff explained the calendar at baseline and collected the calendar at follow-up.

*Toothbrush pump*. Participants received a fluoride toothpaste pump (Colgate Maximum Cavity Protection Pump Fluoride Toothpaste, mild bubble fruit flavored, Colgate-Palmolive Company, New York, NY) during the baseline visit. The pump delivered a controlled amount of toothpaste per episode, and study personnel instructed parents on using the pump.

#### SMS messages

The study sent three types of SMS messages to participants during study weeks. The first message type, sent to all participants every Wednesday, Friday, and Sunday during study weeks, reminded participants to synchronize data from their powered toothbrush to the app. The second message type, sent to intervention and control participants on the Tuesday following each study week, provided feedback on that week’s brushing and syncing performance and provided a summary of earnings that week and cumulative earnings since enrollment. The third message type, sent only to participants in the lottery incentive group on the Sunday prior to the start of the study week, invited participants to select a two-digit number for that week’s lottery drawing. The SMS message bank is provided in S2 Table.

#### Dental visits

Dental visits took place during an initial study visit and the 8-week follow-up visit. During both dental visits, a protocol-trained dental provider who was blinded to treatment arm performed a dental screening on child participant’s teeth (recording decay and fillings), assessed treatment urgency (urgent needs referred with a warm handoff to staff at the corresponding dental clinic), applied liquid plaque disclosing agent, and applied fluoride varnish to the child’s teeth. The plaque assessment and fluoride varnish are described in further detail below.

A boarded pediatric dentist trained the dental providers to perform the visual only screening (no loupes and no explorer) using a standardized protocol where drying of the teeth with gauze was implemented and the dental screenings occurred with a special clinical light in the exam room. However, since dental caries was not a study outcome measure, calibration was not performed. Dental providers followed infection control measures and use of personal protective equipment as a part of the protocol.

Participants in all groups received $30 at the baseline visit and $30 at the follow-up visit as compensation for their time (aside from syncing and incentive earnings).

*Attendance*. Preventive dental visit attendance of participating children was ascertained through dental exam data from the EHS ChildPlus Management Software health module. In the subset of children at baseline without any dental visit in the EHS ChildPlus health module, we assessed whether or not the child had a ChildPlus-documented dental visit by the 8-week follow-up. For purposes of this pilot, feasibility was determined as the ability of study staff to access the EHS software and extract the dental visit information.

*Plaque disclosing solution*. At each in-person visit, the dental provider applied GUM Red-Cote Liquid plaque disclosing liquid agent (Sunstar Americas, Inc., Schaumburg, IL) to the child’s teeth for plaque assessment. A trained research staff member then took extraoral photographs of the facial surfaces of child’s upper incisors with iPhone and Canon cameras (in random order).

*Fluoride varnish*. At each in-person visit, the dental provider applied 5% sodium fluoride varnish with tri-calcium phosphate (Vanish, 3M ESPE, Maplewood, MN) to the teeth to enhance enamel and boost salivary fluoride levels. The varnish contains fluoride, calcium, and phosphate and can be applied quickly without color change to the teeth.

### Measures and milestones

The study’s primary outcome measures and milestones were selected principally to identify the best performing incentive package for a future full-scale trial.

#### Outcomes

The study had two primary outcome measures:

1. The mean number of Bluetooth-recorded half-day episodes per week in which a child’s teeth were brushed during the 8 study weeks. A toothbrushing episode qualified if it lasted ≥1 minute within one of the 14 half-day windows (before or after 12pm) in the week.
2. Dental visit attendance by the 8-week follow-up visit among the subset of children who had no dental visit in the EHS ChildPlus health module at baseline. The toothbrushing incentives were hypothesized to have increased the self-efficacy of parents and their engagement in their children’s oral health care.

Secondary outcomes focused on feasibility and acceptability: willingness of EHS sites to sign a memorandum of understanding; willingness of EHS staff to assist with recruitment; willingness of parents to consent and approve access to EHS ChildPlus dental visit data; willingness of parents to be randomized; willingness of parents to adhere to study procedures; willingness to use the toothpaste pump; willingness to use the powered toothbrush; percent of weeks the participant synced the app; ability to bring the toothbrushing diaries to the follow-up visit; percent of days the participant reported brushing twice based on the parent-reported diaries; change in child toothpaste pump weight; the child’s plaque score (a modification of the Debris Index component on the simplified Oral Hygiene Index, OHI-S [35]) after disclosing with plaque solution; willingness of children to cooperate; and child and parent comfort level with using the powered toothbrush.

*Plaque measurement*. The OHI-S was modified for only the facial surfaces of the four primary maxillary incisor (MI) teeth to rate each tooth surface for plaque using a four-category ordinal score and calculate a mean OHI-MIS score. In addition to having a dental provider rate the plaque during the in-person exam, both an optimized Photomed Canon G16 with ring flash (PhotoMed International, Van Nuys, CA) and iPhone7 (Apple, Cupertino, CA) cameras were used to obtain a photograph of the teeth for asynchronous plaque scoring by a dentist researcher.

#### Milestones

Milestones were set *a priori* as:

1. At least one monetary incentive group (fixed or lottery) will have at least 50% of potential participants hypothetically agree to participate and at least 80% of participants willing to accept randomization to an incentive group (including waitlist (delayed incentive) control).
2. Among groups declared “acceptable,” “feasible,” and “appropriate,” the group with outcomes at least 20% better than the other two groups will be chosen for the future Phase II/III trial. If no group is at least 20% better, then the one that either 1) has the higher toothbrushing performance or 2) is declared most “appropriate” (e.g., easier to explain and understand or less resource intensive) will be chosen for the future Phase II/III trial.

### Sample size

While sample size calculations are generally not required for pilot trials, CONSORT guidelines for pilot trials note that the calculations are appropriate for providing precision and assessing feasibility and acceptability measures [29]. In our case, we designed a pilot study to provide adequate precision and allow comparison of feasibility and acceptability measures among arms. The pilot trial sample size was selected to provide estimates and reasonably precise (narrow) confidence intervals (CIs) of effect sizes for planning a future trial. With 12 dyads per group and *α* = 0.05, the 95% CI for a proportion (e.g., 7 of 14 brushing episodes per week) was estimated to extend 0.100 to 0.283 from the observed proportion depending on the underlying proportion (ranging from 0.032 to 0.968 with 0.5 having the widest interval). In addition, with 3 groups of 12 and *α* = 0.05: (1) a one-way analysis of variance was estimated to have 80% power to detect a difference in percent change means of feasibility and acceptability measures (e.g., willingness to use the Bluetooth-enabled powered toothbrush) when variance of means was 0.024 and a common standard deviation was 0.286; (2) a *χ*^2^ test of feasibility / acceptability measures was estimated to have 80% power to detect a difference in proportions characterized when the variance of proportions of 0.066 and an average proportion of 0.547.

### Data analysis

To estimate each incentive package’s effect on oral hygiene behaviors, we conducted an intention-to-treat (ITT) (as randomized) analysis using a linear mixed-effects model for the number of toothbrushing episodes (identity link) with a random effect for parent-child dyad, fit using restricted maximum likelihood with variance components covariance structure and Kenward-Roger denominator degrees of freedom [36]. Additional specifications adjusted for study week and group × study week effects. We report 95% CIs based on standard errors accounting for the correlation within dyads over time. In exploratory analyses, we estimated linear mixed-effects models of toothbrushing performance, stratified by child age group (≤24 months, 25+ months), because younger children had more behavior problems and anecdotally less comfort with the powered toothbrush.

ITT analyses of toothbrushing performance included data from all dyads that were able to install the Android or iOS S4K app during the baseline visit. Weeks during which a participant did not synchronize any brushing data or recorded zero episodes were classified as having zero qualifying episodes.

We assessed the validity of the toothbrushing measures by evaluating pairwise correlations among Bluetooth toothbrushing data, parent-reported diaries, child plaque scores, and toothpaste usage (change in pump weight). Toothbrushing measures from the smartphone app toothbrushing data and parent-reported diaries were examined overall and by group over time (weekly over 8 weeks) to assess possible temporal effects (e.g., short-term novelty effect of using the powered toothbrush).

McNemar’s *χ*^2^ test was used to compare paired camera feasibility for iPhone vs. Canon photographs for asynchronous plaque rating. Lin’s concordance correlation coefficient with bootstrapped 95% CIs was used to compare in-person vs. asynchronous plaque measures.

We also tallied operational issues with the study products (powered toothbrush, smartphone app, toothbrush pump, SMS messages), reported in the follow-up survey.

Analyses were conducted in SAS (v9.4 Cary, NC) and Stata (v14.2, College Station, TX).

## Results

### Descriptive statistics

**Fig 1:**
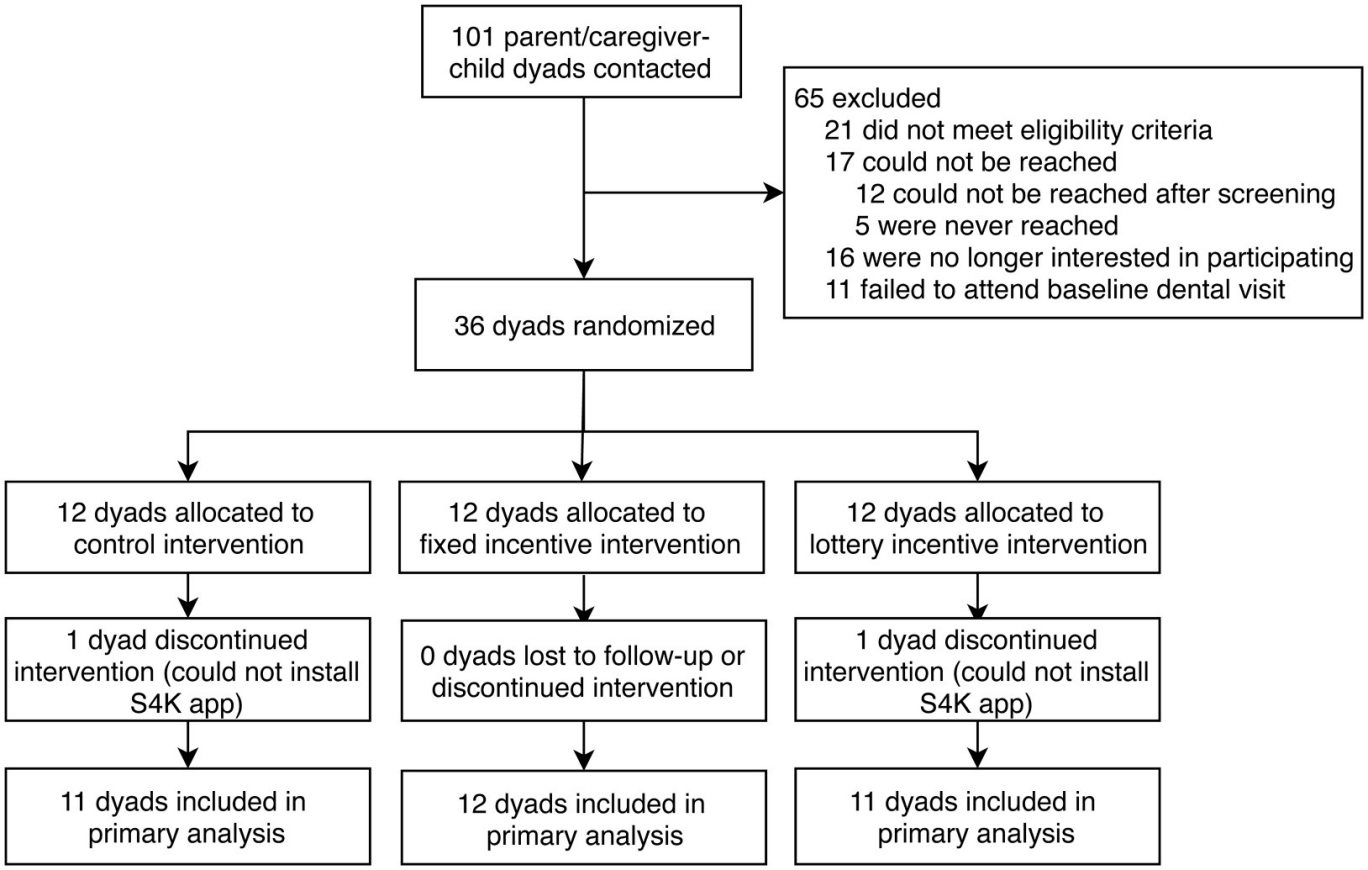
Study flowchart. S4K (Sonicare for Kids) is a Bluetooth-enabled powered toothbrush and accompanying smartphone app.

Fig 1 shows the study flow diagram. In May-July 2017, we recruited 101 parent-child dyads, of whom 36 were eligible and willing to adhere to study procedures, consented and randomized. Two parents could not properly install the Android S4K app during the baseline visit and are excluded from the main analysis. At least 56% of potential participants (57/101) hypothetically agreed to participate in the study, including those who did not meet eligibility criteria and those who consented and participated. All participants agreed to accept randomization to an incentive group. Trial accrual took 39 days, less than the anticipated 56 days (S1 Fig). The study executed memoranda of understanding with an Early Head Start (EHS) program with two sites in Los Angeles County. The study was deemed acceptable to EHS staff, based on participation in study informational sessions and trainings, as well as EHS staff’s willingness to distribute flyers and introduce the program to families for the program during home visits.

Baseline demographic and oral health characteristics were generally balanced across groups (N = 36, Table 1). All caregivers were mothers; about half (14) preferred English to Spanish. About three-quarters stay at home and had household income below the federal poverty line. Thirty-two of 36 participating children were Hispanic/Latinx. Child’s age at enrollment averaged 26.3 ± 8.3 months, (median 26.5, range 9–39), with number of teeth ranging from 2 to 20 (mean ± SD of 16.6 ± 4.8, median 19.0). About 86% of children visited the dentist during the past year (EHS administrative data), as encouraged by EHS. Yet, according to parent self-reports at baseline, only 46% of children had their teeth brushed twice daily for 2 minutes during the prior week and most children (71%) had their teeth brushed daily with fluoride toothpaste.

**Table 1:** Baseline characteristics by study group.

		Study group		
	Total	Control	Fixed incentives	Lottery incentives
	(N = 36)	(N = 12)	(N = 12)	(N = 12)
*Panel A*. *Demographic characteristics*				
Parent is child’s mother	36 (100%)	12 (100%)	12 (100%)	12 (100%)
Prefers English (vs. Spanish)	16 (44%)	3 (25%)	8 (67%)	5 (42%)
Parent graduated high school	20 (57%)	7 (58%)	5 (45%)	8 (67%)
Parent stays at home	28 (78%)	9 (75%)	9 (75%)	10 (83%)
Household income below poverty level	24 (75%)	9 (82%)	7 (70%)	8 (73%)
Child age, in months	26.3 (8.3)	24.5 (8.9)	26.2 (6.5)	28.2 (9.6)
Child is a boy	19 (53%)	9 (75%)	4 (33%)	6 (50%)
Child race/ethnicity				
White, non-Hispanic	2 (6%)	0 (0%)	2 (17%)	0 (0%)
Hispanic or Latino	33 (92%)	12 (100%)	9 (75%)	12 (100%)
Other	1 (3%)	0 (0%)	1 (8%)	0 (0%)
Cell phone is an Android (vs. iPhone)	22 (61%)	8 (67%)	7 (58%)	7 (58%)
EHS site 1 (vs. site 2)	32 (89%)	11 (92%)	11 (92%)	10 (83%)
*Panel B*. *Oral health characteristics*				
No. fully erupted teeth	16.6 (4.8)	15.5 (6.2)	16.9 (3.3)	17.5 (4.7)
Child visited dentist during past year	30 (86%)	9 (82%)	11 (92%)	10 (83%)
Mean plaque index score	2.5 (0.7)	2.5 (0.9)	2.6 (0.5)	2.4 (0.8)
Brush at least twice daily for 2 mins.	16 (46%)	3 (25%)	6 (55%)	7 (58%)
Fluoride used daily	25 (71%)	8 (67%)	6 (50%)	11 (100%)

Data are presented “n (%)” for discrete covariates and “mean (SD)” for continuous covariates.

Study children had high baseline plaque levels on maxillary incisors, despite EHS health education for parents and a high percentage of self-reported parent brushing (80% ≥2 per day) [28]: OHI-MIS scores ranged from 0.5 to 3 (in-person: mean ± SD, 2.5 ± 0.7, median 3.0; asynchronous iPhone 7: 2.6 ± 0.5, median 2.8).

#### Toothbrushing performance

**Fig 2:**
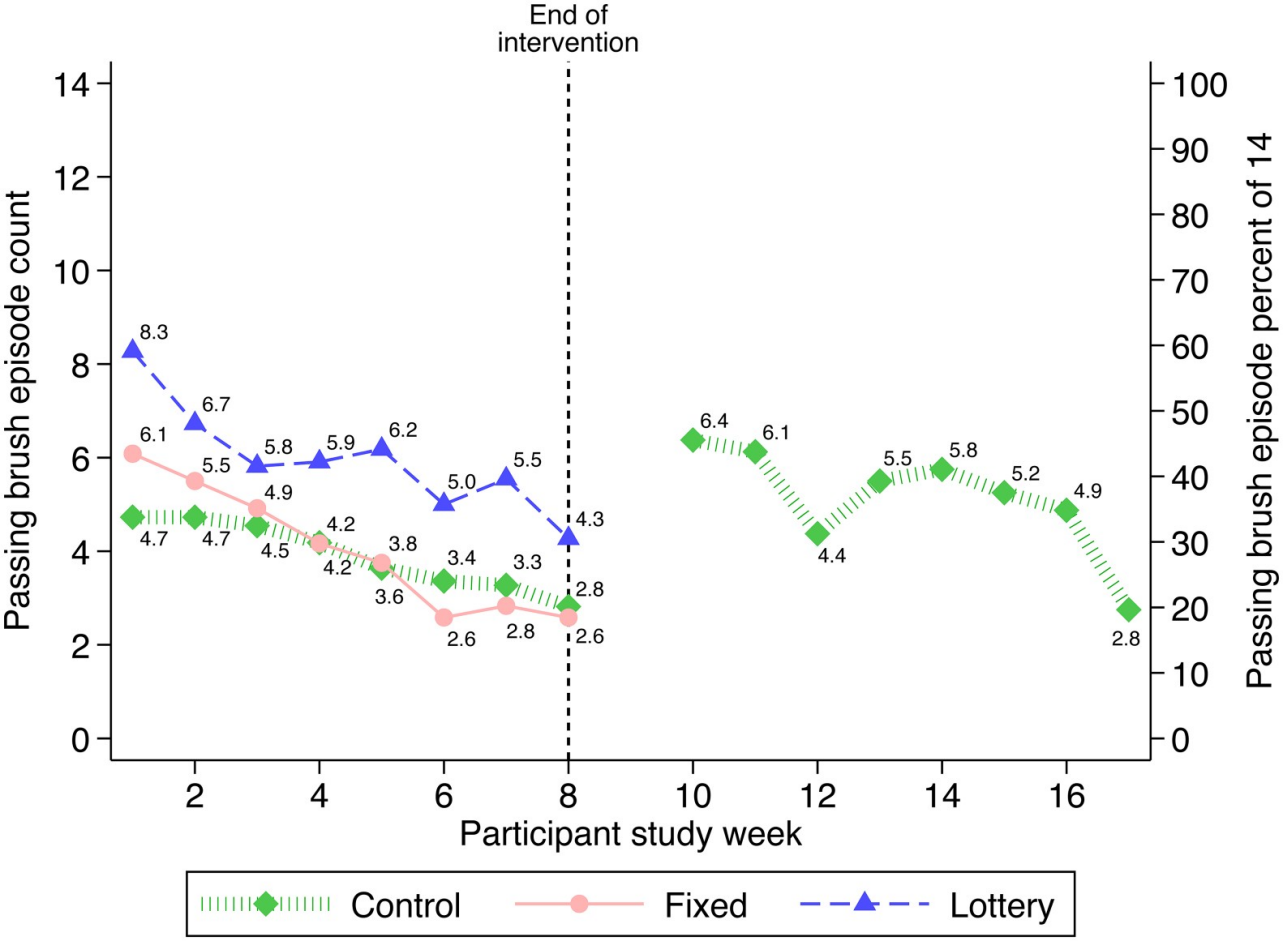
Number and percent (of 14) of toothbrushing episodes by study group and week.

Fig 2 presents average number and percentage (of 14) of qualifying brushing episodes by study week and group. Overall, across all 8 study weeks, participants had a mean ± SD of 4.6 ± 1.1 (median 4.6) qualifying brushing episodes per week. Brushing in the lottery incentive group exceeded the other two groups during each study week. The fixed incentive group and the control group did not differ substantially from each other, although the fixed incentive group started slightly higher during the first couple of study weeks. The mean qualifying episodes declined over time in all three groups.

The waitlist (delayed incentive) control group increased brushing during the delayed incentive period of weeks 10–17, similar to the fixed incentive group during weeks 1–8.

**Fig 3:**
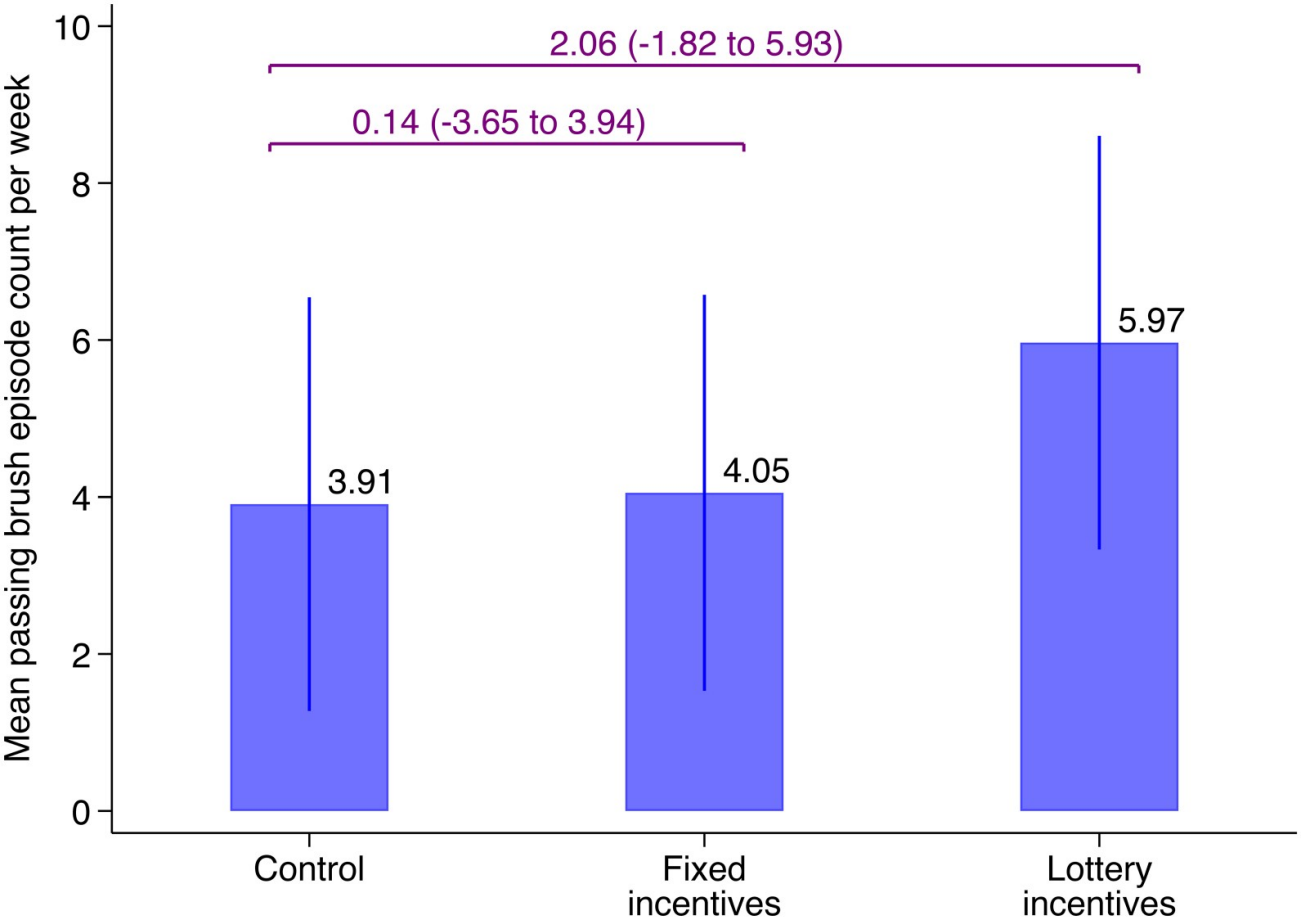
Effects of each incentive package on toothbrushing episodes per week. Linear mixed-effects model with a random effect for dyad. Purple text refers to contrasts of each incentive group against the control group. Error bars represent 95% confidence intervals.

**Fig 4:**
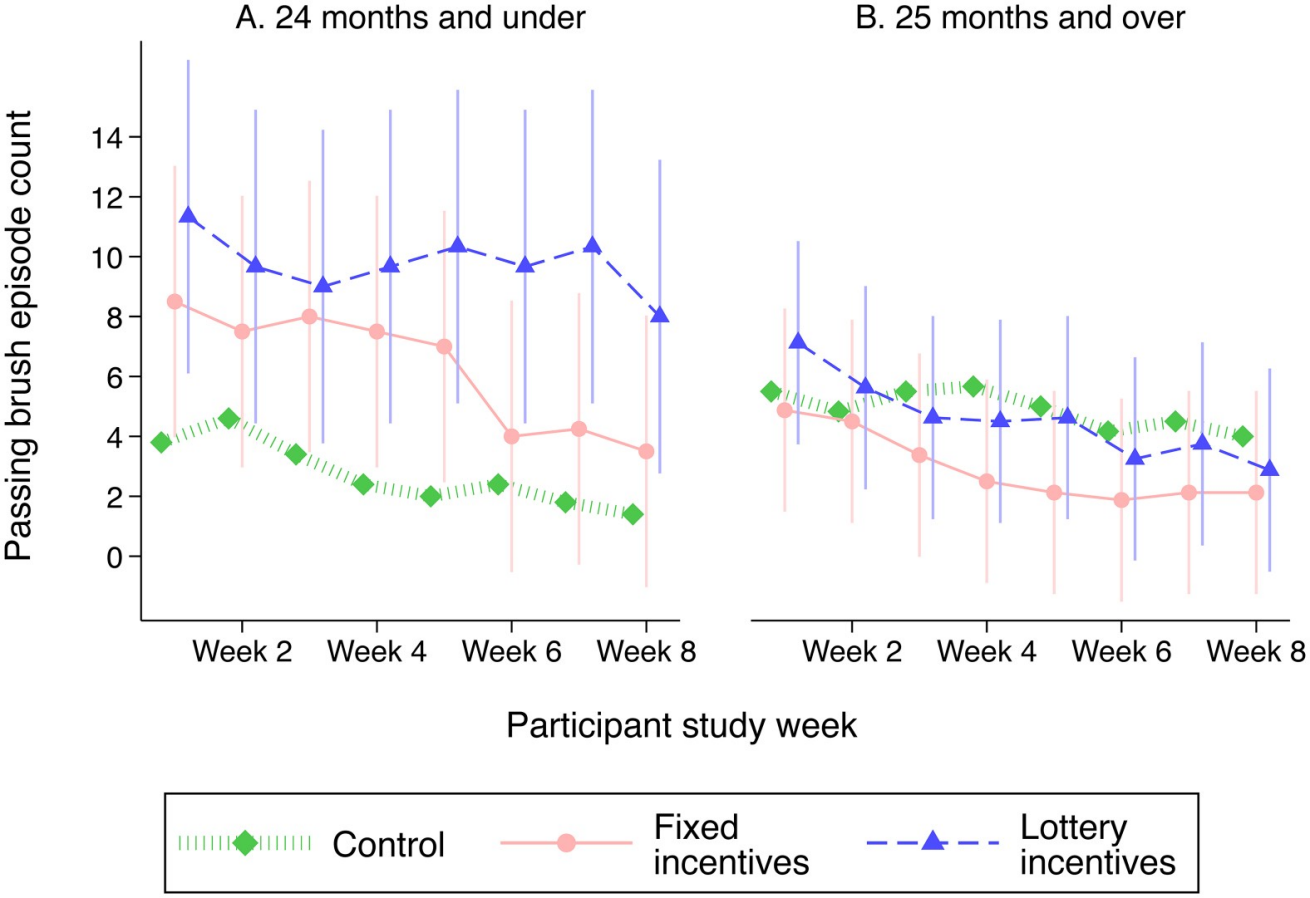
Effects of each incentive package on toothbrushing episodes by study week and child age group. This figure shows a linear prediction of the fixed portion of mixed-effects models with a random effect for child-parent dyad, stratified by child age (≤24 months in Panel A, 25+ months in Panel B). Error bars represent 95% confidence intervals.

Fig 3 shows the main ITT estimates for the primary outcome of toothbrushing per week, from the linear mixed-effects models. The lottery incentive group had more qualifying episodes per week (mean 6.0) than the control group (mean 3.9) (adjusted difference 2.1, 95% CI -1.8 to 5.9). In contrast, the fixed incentive group (mean 4.1) was similar to the control group (adjusted difference 0.1, 95% -3.7 to 3.9). The lottery incentive group had a mean of 1.9 more episodes than the fixed incentive group (95% CI -1.7 to 5.8). Adjusted group differences in mean number and percent correspond to 53% more frequent qualifying toothbrushing for the lottery versus control group and 47% more frequent qualifying toothbrushing for the lottery versus fixed incentive group. In exploratory analyses, we find that the effect of the lottery incentives on qualifying episodes per week is concentrated among children ≤24 months (adjusted differences: fixed vs. control 3.6, 95% CI -2.1, 9.2; lottery vs. control: 7.0, 95% CI 0.8, 13.2), whereas there are no differences in toothbrushing by study group among children ≥25 months (Fig 4 and S3 Fig).

Estimates were similar, adjusting for study week indicators (S3 Table). Plotting a linear prediction of group × study week effects (S2 Fig) yields a pattern very similar to unadjusted results.

Secondary toothbrushing outcomes are presented in Table 2 (Panel B). About 80% of participants used the powered toothbrush during the study. On average, participants synced for 90% of study weeks. About half of parents returned a toothbrushing diary. According to parent-reported diaries, study children brushed twice daily 4.8 days per week on average. Parent-reported diaries and app-recorded episodes per week were modestly positively correlated (r = 0.36, S4 Fig).

**Table 2:** Dental visit attendance and secondary measures by study group.

		Study group		
	Total	Control	Fixed incentives	Lottery incentives
*Panel A*. *Primary dental care outcome*				
Follow-up dental visit among those w/o baseline visit	3/6 (50%)	1/3 (33%)	1/1 (100%)	1/2 (50%)
*Panel B*. *Secondary brushing outcomes*				
Any use of powered brush	28/36 (78%)	9/12 (75%)	9/12 (75%)	10/12 (83%)
Proportion of weeks with any brushing data synced	0.9 (0.1–1.0)	0.9 (0.1–1.0)	0.6 (0.2–1.0)	0.9 (0.1–1.0)
Parent-reported diary completed	19/36 (53%)	5/12 (42%)	7/12 (58%)	7/12 (58%)
Mean days per week with parent-reported twice-daily brushing	4.8 (2.2–6.6)	0.6 (0.4–3.0)	4.4 (2.2–7.0)	6.6 (5.4–7.0)
Change in toothpaste pump weight (grams)	19.6 (12.6–35.4)	13.7 (10.5–16.1)	13.2 (11.9–44.8)	28.8 (21.8–36.4)
Parent comfortable with powered brush	19/25 (76%)	8/8 (100%)	5/7 (71%)	6/10 (60%)
Child comfortable with powered brush	12/26 (46%)	3/9 (33%)	3/7 (43%)	6/10 (60%)
*Panel C*. *Secondary dental care outcomes*				
Consent to central assessment photo	19/36 (53%)	4/12 (33%)	6/12 (50%)	9/12 (75%)
Dental visit at follow-up	24/26 (92%)	7/8 (88%)	8/8 (100%)	9/10 (90%)
Plaque index score at follow-up (in person)	2.5 (1.5–3.0)	2.2 (1.0–3.0)	2.8 (2.5–3.0)	1.8 (1.0–2.5)
Plaque index score at follow-up (iPhone)	2.5 (2.2–3.0)	2.8 (2.2–3.0)	3.0 (2.5–3.0)	2.4 (1.2–3.0)
Child cooperated for baseline dental screening	18/36 (50%)	3/12 (25%)	6/12 (50%)	9/12 (75%)
Child cooperated for baseline disclosing gel application	12/35 (34%)	1/11 (9%)	4/12 (33%)	7/12 (58%)
Child cooperated for baseline plaque image (iPhone)	15/35 (43%)	1/11 (9%)	6/12 (50%)	8/12 (67%)

Data for complete cases are presented “n/N (%)” for categorical variables and “median (interquartile range)” for continuous variables.

Twenty-six participants (72%) returned toothpaste pumps at 8 weeks for weighing. They used a mean ± SD of 28.7 ± 27.5 grams of toothpaste (median 19.6, range 7.6–114.2 grams). Toothpaste usage, measured by the change in toothpaste pump weight, was far higher in the lottery group, consistent with the finding that participants in this group had much higher toothbrushing frequency on average than those in the other two groups. About 76% of parents reported feeling somewhat or very comfortable with the powered toothbrush, compared to 46% reporting their child was somewhat or very comfortable.

**Fig 5:**
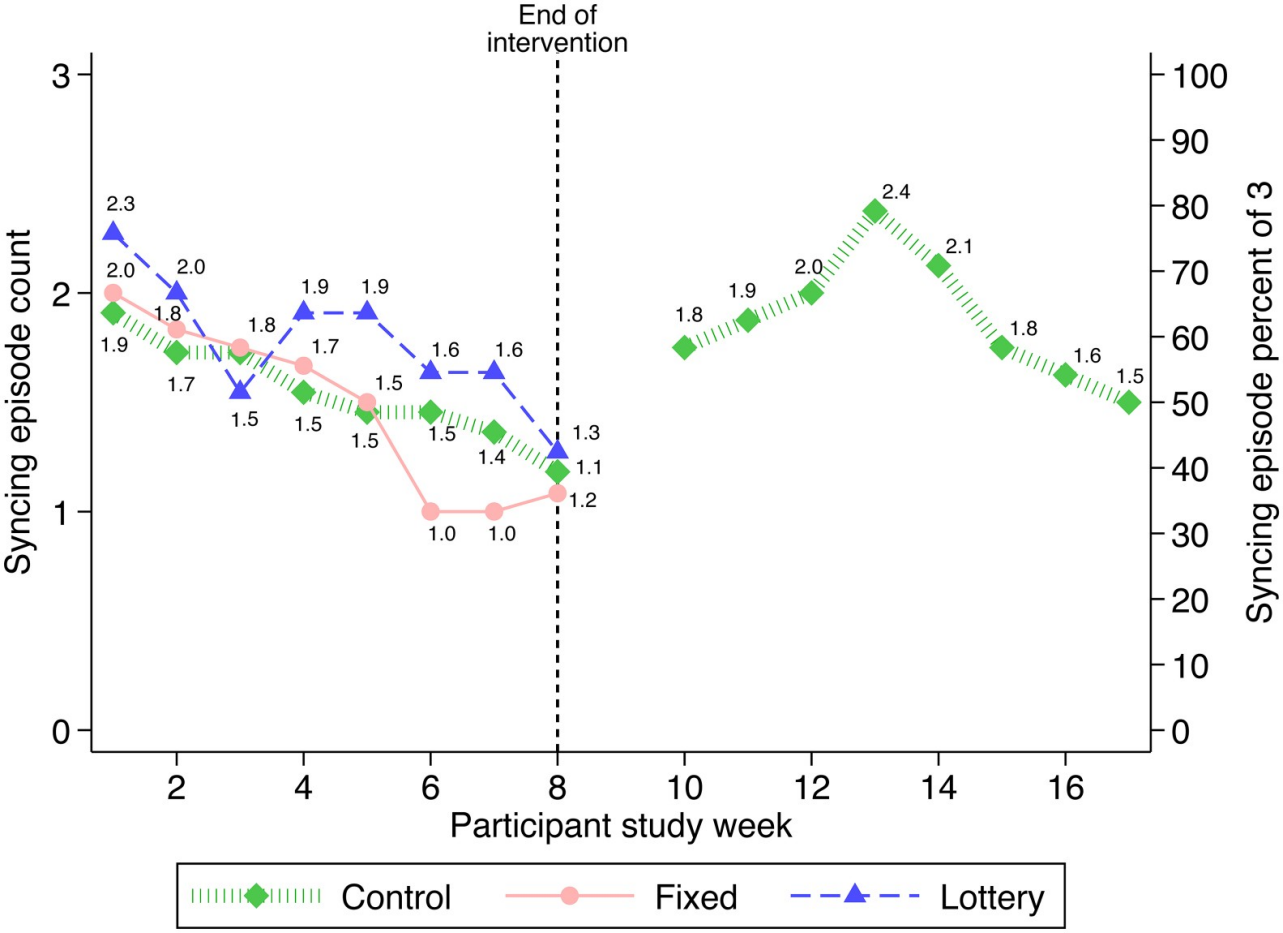
Number and percent (of 3) of syncing episodes by study group and week.

Syncing averaged 1.6 ± 1.3 (mean ± SD) per week (median 2.0). Fig 5 shows mean and percentage (of 3) of syncs per week by group. Most weeks, all three groups had similar means.

### Dental care outcomes

All but six children had a dental visit at baseline. Among the six who did not, three completed a dental visit by the 8-week follow-up (a primary outcome). Overall, 92% of participants who completed the follow-up assessment had a dental visit at baseline or by 8 weeks. Dental visit attendance was similar across groups.

Children had high plaque levels on maxillary incisors (OHI-MIS score) (0–3 range) at the 2-month assessment (in-person: mean ± SD 2.1 ± 1.0, median 2.5; asynchronous iPhone 7: 2.4 ± 0.8, median 2.5). According to the Frankl scale for child behavior, based on the percentage of children who had Frankl scores for child behavior of 1–3 (indicative of cooperation that was not highly negative), child cooperation was 50.0% for baseline dental screening, 34.3% for baseline disclosing gel application, and 42.9% for baseline plaque imaging by iPhone. Frankl scores of child cooperation improved somewhat and exceeded 50% for all procedures during follow-up assessment.

**Fig 6:**
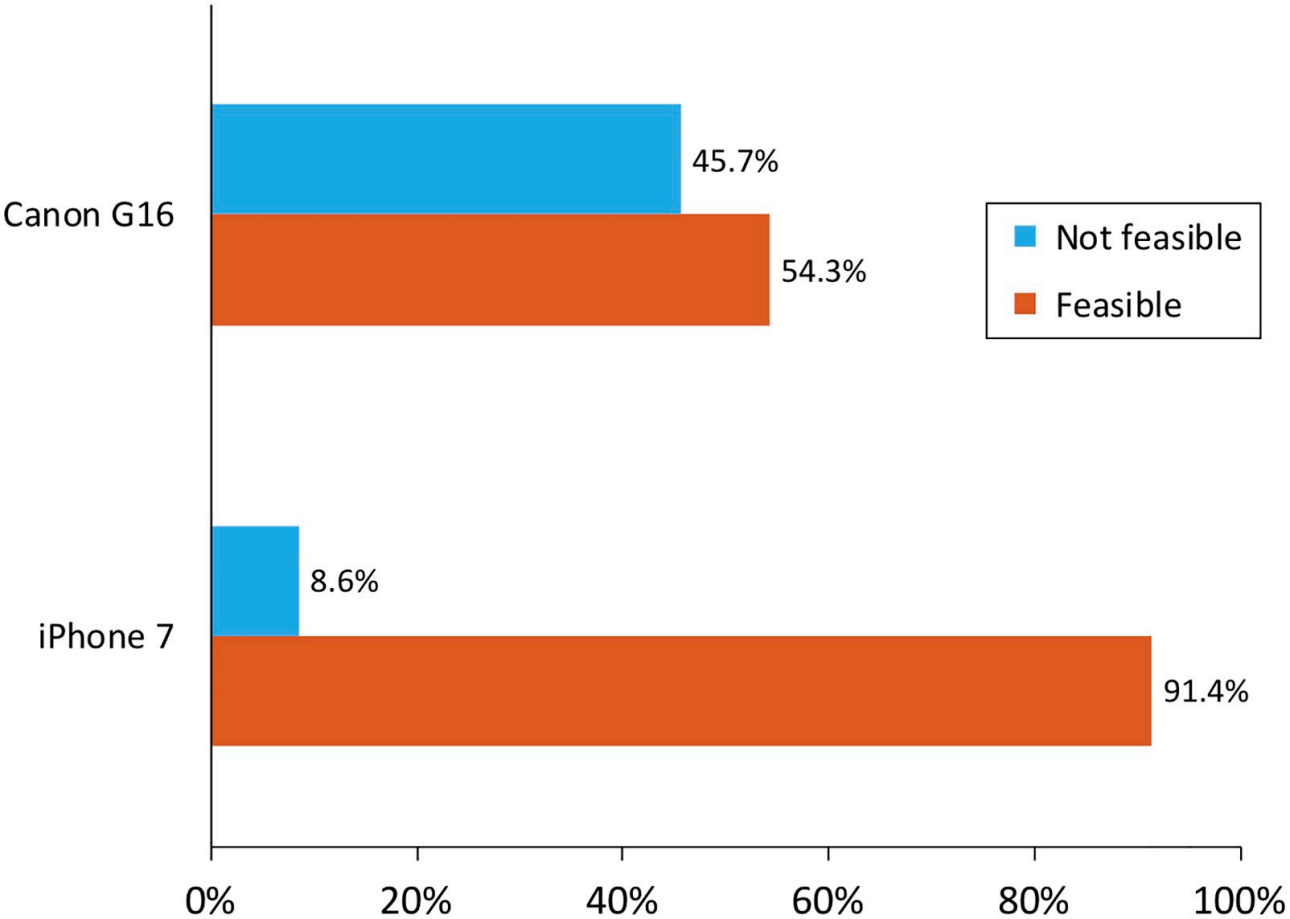
Camera feasibility comparison for photographs for asynchronous plaque rating. McNemar’s test yielded p = 0.0003. The camera order was randomized by participant.

Comparing the two cameras for plaque assessment on maxillary incisors, the iPhone 7 was much more feasible (91%) than the Canon (54%) in producing non-blurry photos (McNemar’s *χ*^2^ test p = 0.0003, Fig 6); camera order/sequence did not seem to matter.

Comparing in-person to asynchronous scoring of images, the in-person OHI-MIS plaque score of the rater who examined most children was highly correlated with the asynchronous plaque score from iPhone 7 photographs (Lin’s concordance correlation of 0.77; bootstrapped 95% CI: 0.41, 0.90).

### Adverse events and operational issues

There were no adverse events reported during the trial. Most parents (80.0%) reported that the powered toothbrush was easy to use. Only one parent reported at follow-up having had any problems with the toothbrush: the vibration was too strong, and the bristles were not soft enough. Most parents (88.5%) found the app to be somewhat or very useful, and all but one child was somewhat or very interested in the app. Seven parents reported at follow-up having had problems with the smartphone app connecting to the powered toothbrush or syncing properly. Nearly all parents (96.2%) were comfortable with using the toothpaste pump, and a similar percentage (92.3%) limited use of the pump to the study child. All parents reported that the number of project SMS messages was “just about right.” Only one parent in the lottery group did not like being asked to select her own lottery numbers.

## Discussion

The BEECON pilot trial demonstrated the feasibility of performing all planned procedures for a large-scale trial, community acceptability of the design and trial procedures, and even proof of concept of monetary incentives to increase toothbrushing monitored through a smart toothbrush synced to an app. Short-term Bluetooth toothbrushing results suggest separation between the incentive and control groups (and perhaps between incentive groups as well). While toothbrushing frequency declined over time across all study groups, the lottery incentive package retained an advantage over the other two groups through 8 weeks. The decline in toothbrushing over time may indicate a novelty effect due to reduced interest in the new product or a Hawthorne (observer) effect [37], although the novelty and Hawthorne effects would have been the same for all three groups.

Both *a priori* milestones were met. More than 50% (56%) of potential participants hypothetically agreed to participate, and more than 80% (100%) of participants were willing to accept randomization to an incentive group. These estimates are hypothetical and inexact, because the trial stopped randomization when the target of N = 36 was met, and potential participants were no longer contacted about return visits. Participants in the lottery incentive package had, on average, 53% more brushing episodes per week than those in the control group and 47% more than those in fixed incentive group. The lottery group, therefore, exceeded the *a priori* milestone of 20% more episodes used to identify an intervention group for a subsequent trial. This increase in toothbrushing frequency, while substantial, showed that many parents still failed to meet the American Academy of Pediatric Dentistry recommendation of twice-daily toothbrushing in children [34], consistent with other studies of toothbrushing frequency in racially/ethnically diverse groups of young children [38]. Our findings may not be comparable to other prior studies, which rely on parent self-reports that are subject to social desirability and recall bias. Our exploratory analyses further suggest that the incentive packages produced the largest improvements in toothbrushing performance among parents with a study child age ≤24 months, who exhibited less cooperative behavior during study interactions with the powered toothbrush.

Health promotion programs often include a form of economic incentives [6, 7, 39]. Recognizing the important role that incentives can play, the US National Institutes of Health has placed evaluating incentives on health-related behaviors among its highest priority areas for health economics research [40]. Incentive structure is critically important to motivating actions. Lottery prizes have been more successful than non-monetary incentives for some health-related behaviors [18, 19, 22, 41], but not others [42, 43]. Differences in lottery structure (e.g., jackpot versus single-tier versus combination) may contribute to these disparate findings [19]. Choosing the best incentive structure and size to motivate sustained behavior change must reflect contextual factors, including interpersonal relations and social preferences. Incentive-based interventions can lead to long-lasting improvements in healthy behaviors, perhaps by fostering the initiation and development of healthy habits and improved self-efficacy that can be sustained even after incentives end [6]. Our study is the first to apply incentive-based approaches to promoting oral health.

### Lessons learned

The pilot trial produced several significant findings with implications for the larger trial currently underway and for future research more generally. We showed the feasibility of adapting the control group into a waitlist (delayed incentive) control group to meet community concerns and to mitigate contamination effects between EHS participants who know one another. This finding led us to adopt a waitlist (delayed incentive) control group for the larger trial.

We used a novel set of digital tools that integrated objective toothbrushing and SMS text data into the REDCap clinical trials management system to measure toothbrushing behavior. The findings suggest that our digital approach would be a feasible and reliable way to collect objective toothbrushing data. Only a small number of participants reported having issues with transmitting their toothbrushing data through the app. Moreover, participants, who received weekly reports via SMS of their Bluetooth-recorded toothbrushing frequency, did not report any discrepancies to us during the trial or in the follow-up questionnaire. A potential concern is that other household members used the study toothbrush handle, although all respondents reported at follow-up that the handle was not shared during the trial, consistent with guidance that parents received during the initial study visit. Objective data on child toothbrushing may be more accurate than parent-reported data [38], and presents opportunities for home-based ECC prevention strategies of the sort tested here.

We built on prior work in behavioral economics to develop fixed and lottery incentive packages, adding a novel design that rewarded differing levels of adherence. We found that a combined (two-tiered) lottery incentive package is a feasible way to promote oral hygiene in young children, similar to a few recent studies that have tested lottery incentives in other health domains [18–22]. A key feature of many of these studies has been the use of digital strategies to remotely monitor and deliver performance feedback for promoting health behavior. Our findings led us to select the lottery incentive package as the intervention arm in the larger trial.

We validated plaque measurement from images in young children using an innovative digital approach that holds potential to characterize individual heterogeneity of treatment effects (a “precision dentistry” effort). Our approach could reduce the burden placed on dental providers to measure plaque in real time, potentially making it easier for them to participate in interventional trials. Based on the feasibility and reliability of the photographic plaque scoring, we will use this approach for outcome measurement in the larger trial.

### Study limitations

This study has several limitations. First, as a pilot trial to test feasibility and acceptability, it was intentionally designed without adequate power to test efficacy of each incentive package. The pilot trial results were used to design a fully powered trial currently underway. Second, the study period was limited to 8 weeks. A longer duration is needed to assess the full effects of each incentive package, especially given the observed decline in number of syncs over the course of the study period. Novelty and Hawthorne effects for powered toothbrushes seem to halt by 12 weeks (e.g., McCracken et al. [44]), but our short-term pilot to assess feasibility, acceptability, and demonstrate proof of concept could not have a 12-week practice run-in period and stay within the time and budget constraints of the developmental/pilot funding mechanism. Third, study participation was limited to parents who own a smartphone and can use smartphone apps and text-messaging. As of 2018, 77% of U.S. adults owned a smartphone, including three-quarters of black and Hispanic adults and two-thirds of adults in low-income households (<$30,000 per year) [45]. Fourth, we could not directly observe the number of times parents synchronized their brushing data to the app, but rather we inferred number of syncs based on whether any Bluetooth episodes were recorded that week. Fifth, weighing toothpaste tubes at the end of the study may not be a true reflection of the toothpaste used for the study child. Because the toothpaste pump controlled the amount of toothpaste per episode, two participants who had well above-average toothpaste usage likely shared the toothpaste with other family members as well. For all other participants, observed toothpaste usage reflected the number of recorded toothbrushing episodes, and the fluoride dose would not pose harm to young children. Finally, the provision of regular feedback reminders to the control group may have led us to understate the true effects of incentives on toothbrushing performance. The reminders were designed to avoid differential under-reporting of toothbrushing episodes in the control group and to isolate the effects of incentive payments themselves. In general, incentives may operate through several mechanisms of action: increasing one’s motivation to engage in the behavior as a result of positive reinforcement; solidifying one’s intentions to perform the behavior; increasing the salience of the behavior; and developing a habit of engaging in the behavior. By providing regular feedback reminders, we may mute the effects of the incentives on increased salience and positive reinforcement in particular. We sacrificed a degree of external validity in order to improve the internal validity of the study.

## Conclusion

In summary, this pilot trial showed for the first time that monetary incentives for oral disease management are feasible, acceptable, and potentially efficacious in young children. Our study highlights the important role that incentive structure can play in influencing toothbrushing behavior, consistent with recent evidence from behavioral economics for other health behaviors. The intervention leveraged multiple digital technologies, including a powered toothbrush, smartphone app, and text-messaging, to deliver the intervention and track participant outcomes. Digital incentive-based programs are a promising strategy that merit further attention from oral health researchers.

## Supporting information

S1 File (PDF)

S1 Checklist (DOC)

S1 Fig Trial accrual. (PDF)

S2 Fig Effects of each incentive package on toothbrushing episodes by study week. This figure shows a linear prediction of the fixed portion of the mixed-effects model with a random effect for child-parent dyad. Error bars represent 95% confidence intervals.
(PDF)

S3 Fig Effects of each incentive package on toothbrushing episodes per week, by child age group. Linear mixed-effects model with a random effect for dyad. Purple text refers to contrasts of each incentive group against the control group. Error bars represent 95% confidence intervals.
(PDF)

S4 Fig Association between parent-reported episodes per week (in diaries) and app-recorded episodes per week. The blue line denotes the regression line, *AppCount* = 4.36 + 0.33*DiaryCount*, *R*^2^ = 0.13. Markers are weighted by frequency of observations.
(PDF)

S5 Fig In-person vs asynchronous plaque rating. Pearson product moment correlation coefficient = 0.769. Lin’s Concordance Correlation Coefficient = 0.765, bootstrap 95% confidence interval (0.407, 0.899).
(PDF)

S6 Fig Cumulative distribution of earnings by study group. Mean cumulative earnings was $30.75 in the fixed incentives group (median $19.50) and $39.82 in the lottery incentives group (median $18.00).
(PDF)

S1 Table Details on lottery drawing using illustrative winning number of 38. The low performance threshold was 7 qualifying episodes per week (roughly one per day). The high performance threshold was 14 qualifying episodes per day (twice per day).
(DOCX)

S2 Table SMS message bank. In the message text, [X] denotes the number of brushing episodes that week; [Y] denotes the number of dollars earned for syncing that week ($0 or $3); [Z] denotes cumulative dollars earned to that point’ and [NM] denotes the two-digit winning lottery number that week.
(DOCX)

S3 Table Effects of incentive packages on number of qualifying brushing episodes. Linear mixed-effects model with a random effect for child-parent dyad and 95% confidence intervals (in parentheses). Model 2 also includes indicators for study week.
(DOCX)

S4 Table Pairwise correlation between toothbrushing measures. This table shows pairwise correlation coefficients between toothbrushing measures. Number of observations for each comparison is in brackets.
(DOCX)

S5 Table Selected secondary outcome measures by age group. Data for complete cases are presented “n/N (%)”.
(DOCX)
