# Supplementary material for: Monetary incentives for improving smartphone-measured oral hygiene behaviors in young children: A randomized pilot trial
Source: PLoS One. 2020 Jul 30;15(7):e0236692. doi: 10.1371/journal.pone.0236692 (PMC7392266; doi:10.1371/journal.pone.0236692)
Supplement: S1 File — (PDF) [file pone.0236692.s001.pdf]

BEECON PILOT TRIAL - Influence of Financial Incentives on Oral Disease Management in Young Children (BEhavioral EConomics for Oral health iNnovation = BEECON)

NIDCR Protocol Number: 16-086-E

NIDCR Grant Number: UH2-DE025514

Principal Investigators: Stuart A. Gansky, DrPH, James G. Kahn, MD, MPH, Francisco Ramos-Gomez, DDS, MS, MPH

NIDCR Program Official: Dena Fischer, DDS, MSD, MS

NIDCR Medical Monitor: N/A

Draft or Version Number: 3.0

03 March 2017

The study will be conducted in accordance with the International Conference on Harmonisation guidelines for Good Clinical Practice (GCP) (ICH E6), the Code of Federal Regulations on the Protection of Human Subjects (45 CFR Part 46), and the NIDCR Clinical Terms of Award. All personnel involved in the conduct of this study have completed human subjects protection training.

SIGNATURE PAGE

The signature below constitutes the approval of this protocol and the attachments, and provides the necessary assurances that this trial will be conducted according to all stipulations of the protocol, including all statements regarding confidentiality, and according to local legal and regulatory requirements and applicable US federal regulations and ICH guidelines.

Principal Investigators or Clinical Site Investigators:

Signed: ______________________________ Date: ______________

Name: Stuart A. Gansky, DrPH

Title: Professor

Signed: ______________________________ Date: ______________

Name: James G. Kahn, MD, MPH

Title: Professor

Signed: ______________________________ Date: ______________

Name: Francisco Ramos-Gomez, DDS, MS, MPH

Title: Professor

TABLE OF CONTENTS

PAGE
SIGNATURE PAGE II
TABLE OF CONTENTS III
LIST OF ABBREVIATIONS VI
PROTOCOL SUMMARY VIII
1 KEY ROLES AND CONTACT INFORMATION 11
2 INTRODUCTION: BACKGROUND INFORMATION AND SCIENTIFIC RATIONALE 13
2.1 Background Information 13
2.2 Rationale 15
2.3 Potential Risks and Benefits 16
2.3.1 Potential Risks 16
2.3.2 Potential Benefits 17
3 OBJECTIVES 18
3.1 Study Objectives 18
3.1.1 Primary 18
3.1.2 Secondary 18
3.2 Study Outcome Measures 18
3.2.1 Primary 18
3.2.2 Secondary 19
4 STUDY DESIGN 21
5 STUDY ENROLLMENT AND WITHDRAWAL 23
5.1 Subject Inclusion Criteria 23
5.2 Subject Exclusion Criteria 23
5.3 Strategies for Recruitment and Retention 23
5.4 Treatment Assignment Procedures 24
5.5 Treatment Assignment Procedures 24
5.5.1 Masking Procedures (if applicable) 24
5.6 Subject Withdrawal 25
5.6.1 Reasons for Withdrawal 25
5.6.2 Handling of Subject Withdrawals or Subject Discontinuation of Study Intervention 25
5.7 Premature Termination or Suspension of Study 25
6 STUDY INTERVENTION 27
6.1 Description of Behavioral Intervention 27
6.2 Administration of Behavioral Intervention 28
6.3 Procedures for Training Interventionists and Monitoring Intervention Fidelity 29
6.4 Assessment of Subject Compliance with Study Intervention 29
7 STUDY SCHEDULE 30
7.1 Screening 30
7.2 Enrollment/Baseline 30

7.3 2-month Follow-Up Study Visit (Visit 2, T = day 60 ± 28) 31
7.4 4-month Follow-up Study Visit (Optional for Control Group Only, Visit 3, T = 120 ± 28) 31
7.5 Withdrawal Visit 32
7.6 Unscheduled Visit 32
8 STUDY PROCEDURES / EVALUATIONS 33
8.1 Study Procedures/Evaluations 33
8.2 Questionnaires 33
8.3 Anticipatory Guidance 33
8.4 Toothbrushing Performance and Frequency 33
8.4.1 Toothbrushing Diaries 33
8.4.2 Smart Toothbrushes 33
8.5 Dental Visit Attendance 34
8.6 Study Products 34
8.6.1 Plaque Disclosing Solution 34
8.6.2 Fluoride Varnish 34
8.6.3 Fluoride Toothpaste Pump 34
9 ASSESSMENT OF SAFETY 35
9.1 Specification of Safety Parameters 35
9.1.1 Unanticipated Problems (UPs) 35
9.1.2 Adverse Events 35
9.1.3 Serious Adverse Events (SAEs) 36
9.2 Time Period and Frequency for Event Assessment and Follow-Up 36
9.3 Characteristics of an Adverse Event 36
9.3.1 Relationship to Study Intervention 36
9.3.2 Expectedness of AEs 37
9.3.3 Severity of Event 37
9.4 Reporting Procedures 37
9.4.1 Unanticipated Problem (UP) Reporting to IRB and NIDCR 37
9.4.2 Serious Adverse Event Reporting to IRB and NIDCR 38
9.4.3 Reporting of SAEs and AEs to FDA 39
9.4.4 Events of Special Interest (if applicable) 39
9.5 Halting Rules 39
10 STUDY OVERSIGHT 41
11 CLINICAL SITE MONITORING 42
12 STATISTICAL CONSIDERATIONS 43
12.1 Study Hypotheses 43
12.2 Sample Size Considerations 43
12.3 Final Analysis Plan 43
13 SOURCE DOCUMENTS AND ACCESS TO SOURCE DATA/DOCUMENTS 45
14 QUALITY CONTROL AND QUALITY ASSURANCE 46
15 ETHICS/PROTECTION OF HUMAN SUBJECTS 47

15.1 Ethical Standard 47
15.2 Institutional Review Board (IRB) 47
15.3 Informed Consent Process 47
15.4 Exclusion of Women, Minorities, and Children (Special Populations) 48
15.5 Subject Confidentiality 48
16 DATA HANDLING AND RECORD KEEPING 49
16.1 Data Management Responsibilities 49
16.2 Data Capture Methods 49
16.3 Schedule and Content of Reports 49
16.4 Study Records Retention 49
16.5 Protocol Deviations 50
17 PUBLICATION/DATA SHARING POLICY 51
17.1 Peer Review 51
17.2 Authorship 51
18 LITERATURE REFERENCES 53
SUPPLEMENTAL MATERIALS 59
APPENDICES 60
APPENDIX A: SCHEDULE OF EVENTS 60

LIST OF ABBREVIATIONS

AE	Adverse Event/Adverse Experience
CAPI	Computer Assisted Personal Interview
CC	Coordinating Center
CFR	Code of Federal Regulations
CHR	Committee on Human Research
CI	Confidence Interval
CRF	Case Report Form
CRO	Contract Research Organization
CTMS	Clinical Trials Management System
DHHS	Department of Health and Human Services
DI	Debris Index
dmfs	Number of decayed, missing, and filled tooth surfaces
ECC	Early Childhood Caries
eCRF	Electronic Case Report Form
EHS	Early Head Start
FDA	Food and Drug Administration
GCP	Good Clinical Practice
ICH	International Conference on Harmonisation
ICMJE	International Committee of Medical Journal Editors
IRB	Institutional Review Board
N	Number (typically refers to subjects)
NIDCR	National Institute of Dental and Craniofacial Research, NIH, DHHS
NIH	National Institutes of Health
OCTOM	Office of Clinical Trials Operations and Management, NIDCR, NIH
OHI	Oral Hygiene Index
OHRP	Office for Human Research Protections
PHI	Protected Health Information
PI	Principal Investigator
POQL	Pediatric Oral Health Quality of Life
SAE	Serious Adverse Event/Serious Adverse Experience
TB	Toothbrush
TP	Toothpaste

UP	Unanticipated Problem
US	United States

PROTOCOL SUMMARY

Title: BEECON PILOT TRIAL - Influence of Financial Incentives on Oral Disease Management in Young Children (BEhavioral EConomics for Oral health iNnovation = BEECON)

Précis: NIH has placed the evaluation of incentives on health-related behaviors among its highest priority areas for health economics research (NIH, 2016). Incentive structure and size are critical to motivating actions and may result in highly context-dependent effects.

This Phase I pilot trial will: a) test two intervention regimens versus a control regimen to determine which intervention group will be used for the future Phase II/III trial, and b) finalize the outcome measures to be used in that future trial. Overall, we hypothesize that one micro-incentive strategy informed by behavioral economic theory will be more promising than the control and the other strategy for promoting preventive dental care behaviors by parents/caregivers for their children under 3.5 years old.

Objectives: Primary: The primary objective of this pilot trial is to assess the feasibility and acceptability of the interventions and trial design implementation for two micro-incentive reward programs (denoted as “fixed payment” and “drawing payment”) versus a control program in predominantly Latino parents/caregivers to increase their early childhood caries (ECC) preventive health behaviors (i.e., toothbrushing adherence and dental visit attendance) with their children enrolled in/waitlisted for Early Head Start (EHS) home visit programs.

Secondary: Secondary objectives of the pilot trial are to
(1) Finalize primary outcome measures of parent/caregiver behaviors for their children’s oral health, for the future Phase II/III trial:

- (a) Assess feasibility/acceptability and concordance of toothbrushing performance and frequency measures, which will be used to assess parent/caregiver adherence with children’s twice daily toothbrushing using fluoride dentifrice;

	(b) Assess feasibility of obtaining dental exam data from Head Start's ChildPlus Management Software health module, which will be used to collect preventive dental visit attendance of participating children under 3.5 years old; and<br>(2) Define secondary outcome measures of parental/caregiver behaviors for their children's oral health promotion and disease management, for the future Phase II/III trial, including child dental screening treatment urgency; parent/caregiver knowledge, attitudes, and beliefs about preventive oral healthcare (for both home management and clinical care); parent/caregiver-reported health behaviors related to their own oral health (e.g., toothbrushing); and reported preventive behaviors for other children and adults in the family.
**Population:**	Parent/caregiver-child dyads (6 months to 3.5 years) enrolled in or waitlisted for an Early Head Start home visit program in Los Angeles, CA (n=36 dyads)
**Number of Sites:**	Up to 3 Los Angeles County Early Head Start Centers
**Description of Intervention:**	The trial will compare 3 groups that will offer a fixed monetary incentive package, drawing monetary incentive package, or non-monetary incentive (waitlisted control group) for toothbrushing adherence and dental visit attendance.
**Study Duration:**	2-3months
**Subject Participation Duration:**	Parent/caregiver and child dyads: 2 study visits, ~2 months apart
**Estimated Time to Complete Enrollment:**	1.5 months

Schematic of Study Design:

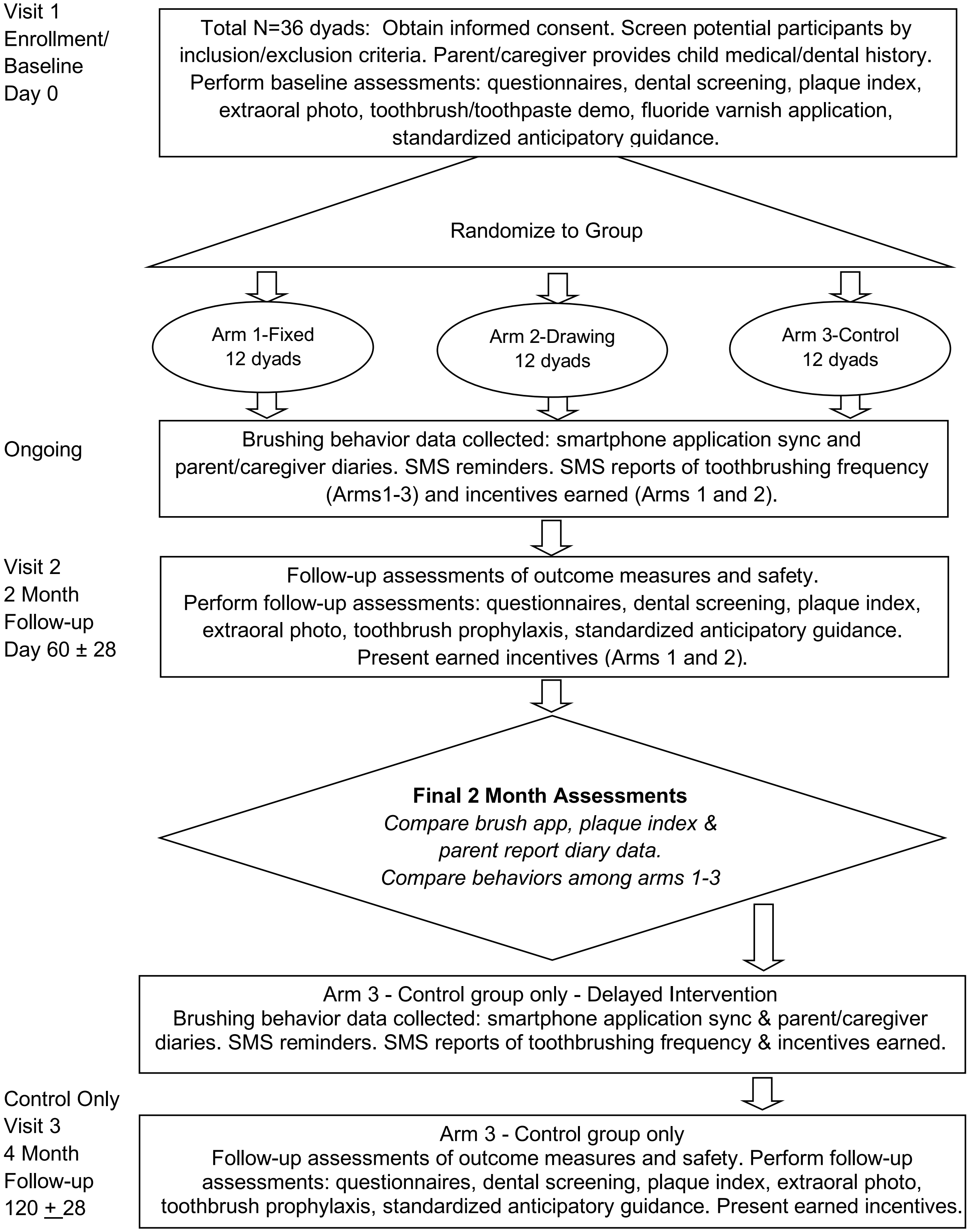

1 KEY ROLES AND CONTACT INFORMATION

**Principal Investigators:**	Stuart A. Gansky, DrPH<br>University of California, San Francisco<br>Box #1361<br>San Francisco, CA 94143<br>tel: 1-415-502-8094<br>fax: 1-415-502-8447<br>stuart.gansky@ucsf.edu
	James G. Kahn, MD, MPH<br>University of California, San Francisco<br>Box #0936<br>San Francisco, CA 94143<br>tel: 1-415-476-6642<br>fax: 1-415-476-0705<br>JGKahn@ucsf.edu
	Francisco Ramos-Gomez, DDS, MS, MPH<br>University of California, Los Angeles<br>10833 Le Conte Ave.<br>Box 951668<br>Los Angeles, CA 90095-1668<br>tel: 1-310-825-9460<br>fax: 1-310-206-7597<br>frg@dentistry.ucla.edu
**NIDCR Project Scientist:**	Dena Fischer, DDS, MSD, MS<br>National Institute of Dental and Craniofacial Research (NIDCR)<br>6701 Democracy Blvd, MSC 4878<br>Bethesda, MD 20892-4878<br>tel: 1-301-594-4876<br>dena.fischer@nih.gov
**Institutions:**	Venice Family Clinic Early Head Start<br>2509 Pico Blvd<br>Santa Monica, CA 90405-1828
**Other Key Personnel:**	Jenny X. Liu, PhD<br>University of California, San Francisco<br>Box #0646<br>San Francisco, CA 94143<br>tel: 1-415-502-5200<br>fax: 1-415-502-5208<br>Jenny.Liu2@ucsf.edu

Justin S. White, PhD
University of California, San Francisco
Box #0936
San Francisco, CA 94143
tel: 1-415-476-8045
fax: 1-415-476-0705
Justin.White@ucsf.edu

Bonnie Jue, DDS
University of California, San Francisco
Box #1361
San Francisco, CA 94143
tel: 1-415-305-9701
fax: 1-415-502-8447
bonnie.jue@ucsf.edu

2 INTRODUCTION: BACKGROUND INFORMATION AND SCIENTIFIC RATIONALE

2.1 Background Information

ECC is a Public Health Problem: Rationale for Prevention Strategies. Early childhood caries (ECC) remains the most prevalent chronic childhood disease in the United States (Vargas et al., 1998; Poland & Hale, 2003;CDC, 2001) and poses a serious threat to child welfare, particularly among economically disadvantaged, underserved, and migrant children (Ramos-Gomez et al., 2007; DHF, 2006). National ECC prevalence (any decayed, extracted, or filled primary teeth (deft) among 2–5 year olds) increased from 24% in 1988–1994 to 28% in 1999–2004 (Dye et al., 2007) but decreased to 23% in 2011-2012 (Dye et al., 2015). Mexican-Americans were 3.5-4.6 times more likely to have ECC than the general US population (Grim et al., 1994). Untreated caries in primary teeth has been associated with pain, difficulty eating, speech impediment and being underweight (Vargas et al., 1998).

Furthermore, longitudinal studies report that preschool-age children with ECC have higher risk for caries and dental problems in permanent teeth later in life, affecting psychosocial well-being, growth, and development (Greenwell et al., 1990; Kaste et al., 1992; Reisine et al., 1994; O’Sullivan & Tinanoff, 1996; Matilla et al., 1998; Peretz et al., 2003).

Restoration of dentition is expensive, and estimated costs to treat children increase with greater disease severity. For example, when decayed, extracted or filled teeth is 18, average costs are estimated to be $1725 (Ramos-Gomez et al., 1996); anesthesia and/or sedation (often required) adds $50-510 per session (Ramos-Gomez, 2015 unpublished data). Private practices generally cost more than public clinics; “for example, in Los Angeles [in 2012], the midpoint fee charged by local dentists for a tooth extraction was nearly three times the full fee charged by a health center serving residents of the same community.” (GAO, 2013).

Initiating early preventive care measures may contribute to maintenance of children’s oral health (Ramos-Gomez, 2014). The American Dental Association (ADA) and American Academy of Pediatrics (AAP) both recommend infants see a dentist by age one, or within six months of first tooth eruption (ADA, 2014; AAP, 2003). Consuming fluoridated drinking water during infancy fosters healthy teeth (AAP, 2003). Since dental disease risk increases as a child’s teeth erupt (ADA, 2014), brushing with a smear of fluoridated toothpaste (TP) for two minutes twice daily (Creeth et al., 2009) can help to prevent ECC. For 1 to 2 year old children, professional application of fluoride varnish (FV) over 2 years is efficacious prevention (Weintraub et al., 2006; Gansky et al., 2007).

Types of Micro-incentives. Incentives are a common component of health promotion programs. In recognition of the important role that incentives can play, NIH has placed the evaluation of incentives on health-related behaviors among its highest priority areas for health economics research (NIH, 2016). Incentive structure and size are critically

important to motivating actions and may result in highly context-dependent effects. A few studies have directly compared different incentive modalities for promoting preventive behaviors (Deren et al., 1994; Kamb et al., 1998; Laken & Ager, 1995; Malotte et al., 1999; Melnikow et al., 1997; Smith et al., 1990; Curry et al., 1991; Wing et al., 1996). Individuals prefer monetary payouts to non-monetary equivalents for HIV/STD and tuberculosis screening (Deren et al., 1994; Kamb et al., 1998; Malotte et al., 1999). Lottery prizes have been more successful than non-monetary incentives for encouraging more physical activity (Wing et al., 1996), smoking cessation, and blood donation (Dey et al., 1999; Goette & Stutzer, 2008), but not for encouraging prenatal visit attendance (Laken & Ager, 1995). Non-monetary incentives (e.g., social recognition, gifts) have effectively boosted immunization (Banerjee et al., 2010; Birkhead et al., 1995; Nexoe et al., 1997; Satterthwaite, 1997) and follow-up cancer screening visits (Marcus et al., 1992; Mayer & Kellogg, 1989), and non-monetary incentives may work as well as material gifts especially if aimed at reducing barriers (e.g., transportation) (Melnikow et al., 1997; Smith et al., 1990). Hence, it is unclear which modality (e.g., fixed versus drawing payment) may most effectively promote positive oral health behaviors.

Incentive size is also important, in complex ways. Although increasing cash and voucher amounts increase behavioral responses for smoking cessation (Malotte et al., 1998; Stitzer et al., 1986), substance abuse therapy (Lussier et al., 2006), and weight loss (Finkelstein et al., 2007), the largest incremental effect is sometimes associated with the smallest initial amount (Thornton, 2008) and excessive values may even have unintended consequences. For example, large incentives may displace intrinsic motivation for behavior change with the expectation of extrinsic rewards to achieve the same health goals, and consequently remove motivation to adhere to new behaviors once rewards are withdrawn (Gneezy & Rustichini, 2000; Della Vigna & Malmendier, 2006). Similarly, incentives may alter expectations of interpersonal trust (e.g., between patients and doctors) if exchanges with incentives are viewed as monetary transactions (Heyman & Ariely, 2003), and discourage altruism if they conflict with intrinsic altruistic motivations (Benabou & Tirole, 2003; Benabou & Tirole, 2006; Lacetera & Macis, 2010).

Choosing the best incentive form and size to motivate sustained behavior change must reflect contextual factors, including interpersonal relations and reciprocity. Cash may be preferred when individuals evaluate options over only one dimension (i.e., monetary value), but when affective dimensions are added (e.g., working toward a collective goal) non-monetary incentives may perform better (Shaffer & Arkes, 2009). The perceived value and interpretation of the incentive payout may differ across populations and cultures (e.g., for study recruitment and retention); Americans may differ by race/ethnicity in their preferences for monetary and non-monetary incentives (Lee & Cheng, 2006). Some African Americans have preferred a nominal cash payout over a gift equivalent (Herring et al., 2004), and Latino Americans have responded variably to vouchers compared to cash (Ramos-Gomez et al., 2008; Martinez-Ebers, 1997). Cash sometimes elicits greater responses among groups with lower education levels (Petrolia & Bhattacharjee, 2009), but nonworking respondents may respond better to in-kind gifts (Ryu et al., 2006).

Populations place different value on the reward associated with different incentive types (i.e., if the reward is viewed simply as a commercial transaction versus as an added symbolic value of interpersonal trust (vis-à-vis social exchange theory) (Gneezy & Rustichini, 2000; Homans, 1974; Burns, 1973).

Lastly, incentive payout form and size affect program sustainability. While conditional cash transfer programs successfully alleviate poverty and promote social goals, they are expensive (Handa & Davis, 2006). Moreover, policy makers may prefer in-kind to cash transfers (Das et al., 2005). For the same cost, differently structured incentives may have substantially different effects (Volpp et al., 2009; Haisley et al., 2012). Although some studies have included a cost-effectiveness calculation to evaluate an incentive's added value (Kane et al., 2004), none have done so for oral health.

The project's overall goal is to assess the efficacy of micro-incentives towards predominantly Latino parents/caregivers to promote ECC preventive health behaviors (dental visit attendance and toothbrushing adherence) of their children in EHS home visit programs.

This protocol describes a pilot trial, the purpose of which is to assess feasibility and acceptability of the interventions and trial design implementation being considered for the future Phase II/III trial, finalize parent/caregiver behavior change primary outcome measures (adherence to brushing child's teeth twice per day with fluoridated TP and timely dental visit attendance), and define secondary outcome measures for parent/caregiver behavior and disease management.

2.2 Rationale

Rationale for Micro-incentives. Despite infant oral care programs' efficacy (Savage et al., 2004; Lee et al., 2006; Rozier et al., 2003), several obstacles impede early preventive dental care. Many dentists do not accept patients younger than age three (Seale & Casamassimo, 2003), and many pediatric dentists do not accept government dental insurance (e.g., Medicaid) (Pourat et al., 2014; Kranz et al., 2014). Numerous factors may contribute to parents/caregivers' inability to provide ADA- or AAP-recommended oral health prevention for their children (i.e., consume fluoridated water, brush with a smear of fluoridated TP for 2 minutes twice daily, and attend regular oral health appointments by age one or within six months of first tooth eruption). Minimal oral health knowledge and/or literacy, financial obstacles, and competing influences, such as daily stress, work, housing priorities, childcare needs, transportation, or geographic location, are a few considerations that may result in failure to follow these oral health recommendations. Further, cultural influences may lead to distrust of fluoride, or believing preventive oral care is unnecessary (Butani et al., 2008). For example, at UCLA's Infant Oral Care Prevention Clinic, retention for 0-5 year olds treated for caries was only 35% (Ramos-Gomez, unpublished data).

Micro-incentives have facilitated positive changes in health-promoting behaviors, such as quitting smoking (Marteau et al., 2009; Rogers et al., 2014), weight loss (John et al., 2011), and managing blood glucose and blood pressure (Sen et al., 2014). Conditional rewards are particularly effective in encouraging behavioral changes; in some cases small incentives can be equally effective or more effective than larger ones (Marteau et al., 2009; Sen et al., 2014). These "behavioral economic" interventions can lead to healthy behaviors even after incentives end, particularly with smaller incentives (Sen et al., 2014). To date, no study has evaluated such a program for oral health.

2.3 Potential Risks and Benefits

2.3.1 *Potential Risks*

This study poses minimal risk to participants. Study participation is completely voluntary, and participants may discontinue participation at any time without prejudice. Regarding study questionnaires, parent participants may experience psychological discomfort in discussing personal health information or negative experiences at a dental or other relevant office (such as enrolling a child in Medicaid). As with any study, there is the potential for loss of confidentiality. Appropriate precautions will be taken to mitigate this risk. These include the use of unique study codes for participants and password-protected computers for data storage. Compliance with all Institutional Review Board (IRB) regulations concerning data collection, data storage, and data destruction will be strictly observed. Data will only be accessible to research personnel and will be stored and coded according to guidelines set forth by the UCSF CHR which is the overseeing IRB.

Even though it is extremely unlikely that a child will get access to and eat the TP, using TP pumps will further reduce the chance of toxicity since the pumps require dexterity that children in the study age range likely lack, and the pumps limit the amount of toothpaste dispensed at any one time. Parents will be advised to keep the toothpaste on a counter and out of the reach of all children in the household.

There is a possibility that a participant may experience an allergic reaction to the TP, fluoride varnish, and/or plaque disclosing solution; however, the likelihood from TP and FV is extremely rare, while the likelihood from disclosing solution is also rare according to the package insert. Parents/caregivers will be instructed to contact the study's Program Director in the unlikely event that an allergic reaction to a study product is suspected.

Any adverse reactions will be reported as outlined in Section 9.

All parent/caregiver participants will be asked if they are willing to be contacted again for research purposes. For those who agree, personal identifying information (PII), including contact information, will be requested for subsequent contact and interviewing, creating the risk of a loss of confidentiality of contact information. All participants will be informed of this risk and of steps taken to reduce this possibility.

2.3.2 *Potential Benefits*

Each child participating in this pilot trial, regardless of study group, will have a dental screening and fluoride varnish applied to his/her teeth. Each child participant will also receive oral hygiene aids, such as a “smart” powered toothbrush and fluoride TP pumps. Children will be provided with referrals for dental visits as needed to providers who agree to take their insurance. In addition, parents/caregivers will be provided Anticipatory Guidance in a standardized video format in English or Spanish; families participating in this study may benefit from the Anticipatory Guidance information.

Overall, participants in the study may benefit from an increased awareness of oral health prevention behaviors.

3 OBJECTIVES

3.1 Study Objectives

This Phase I pilot trial will test two intervention regimens versus a control regimen to determine which intervention will be used for the future Phase II/III trial and will finalize the outcome measures to be used in the future trial.

3.1.1 *Primary*

The primary objective of this pilot trial is to assess feasibility and acceptability of the interventions and trial design implementation for two micro-incentive reward programs (denoted as “fixed payment” and “drawing payment”) versus a control program in predominantly Latino parents/caregivers to increase ECC preventive health behaviors (i.e., toothbrushing adherence and dental visit attendance) of their children enrolled in/waitlisted for EHS home visit programs.

3.1.2 *Secondary*

The secondary objectives of the pilot trial are to:

1) Finalize primary outcome measures of parent/caregiver behaviors for their children’s oral health, for the future Phase II/III intervention study:
    a. Assess feasibility/acceptability and concordance of toothbrushing performance and frequency measures, which will be used to assess parent/caregiver adherence with children’s twice daily toothbrushing using fluoride dentifrice;
    b. Assess feasibility of obtaining dental exam data from Head Start’s ChildPlus Management Software health module, which will be used to collect preventive dental visit attendance of participating children.
2) Define secondary outcome measures of parental/caregiver behaviors for their children’s oral health promotion and disease management, for the future trial, including child dental screening treatment urgency; parent/caregiver knowledge, attitudes, and beliefs about preventive oral healthcare (for both home management and clinical care); parent/caregiver-reported health behaviors related to their own oral health (e.g., toothbrushing); and reported preventive behaviors for other children and adults in the family.

3.2 Study Outcome Measures

3.2.1 *Primary*

Acceptability and feasibility of the fixed payment and drawing payment interventions will be measured with the following outcome measures: a) the percentage of half-days (i.e., mornings, evenings) during the study observation period that a parent/caregiver brushes his/her child’s teeth and b) in the subset of children at baseline with no dental visit in the EHS ChildPlus health module, whether or not the child has a ChildPlus documented

dental visit at the three-month follow-up. The feasible and acceptable intervention that yields better toothbrushing adherence and dental attendance outcomes will be selected for inclusion in the future trial.

Additional primary outcome measures of the trial intervention include participant acceptability and feasibility measures in the EHS organization, and amongst EHS staff, parent/caregivers and children. Measures include willingness of the following:

- LA County EHS to sign memoranda of understanding;
- EHS staff participation in assisting with recruitment;
- parents/caregivers to provide informed consent and approve access to ChildPlus dental visit data;
- parents/caregivers to be randomized to a study arm;
- parents/caregivers to adhere to study procedures, including completing questionnaire instruments and toothbrushing frequency diaries, bringing toothpaste pump to 2mo follow-up visit; and
- child cooperation with study procedures, including dental screening, extraoral photo and plaque assessment.

Additional acceptability and feasibility measures of the trial intervention will include meeting inclusion criteria (smartphone ownership and SMS texting), willingness to use the products provided, including the TP pump and child and parent comfort level with using the smart, powered toothbrush and privacy concerns related to tracking their toothbrushing habits.

3.2.2 *Secondary*

To finalize the primary outcome measures of parent/caregiver oral health behaviors for their children, the following secondary measures will be collected.

Twice daily brushing with fluoridated TP will be ascertained with the following measures, and concordance will be assessed:

- parent/caregiver-reported frequency diaries;
- child's smart powered toothbrush (TB) recorded data;
- change in child TP pump weight; and
- the child's Debris Index component (plaque score) of the Oral Hygiene Index (Greene and Vermillion) after disclosing with plaque solution.

Feasibility of measuring toothbrushing frequency with diaries will include bringing toothbrushing diaries to the follow-up visit and willingness to provide them to study staff. Feasibility of using TP pump weight as a measure of usage will be remembering to bring the TP pump to the follow-up visit for weighing. Feasibility of using the disclosing solution will be measured by the willingness of children to comply with the procedure. Feasibility of using photographs for central assessment will be the willingness of the child to allow

photographs, the ability to transmit the photographs securely, and the ability of the central clinician to score them reliably and confidently.

Feasibility of the smart powered TB handle will be willingness and ability to use the smart powered TB and sync the TB with the app. Additionally, to understand technology usability issues, survey questions at follow-up will ask about any issues parents/caregivers encountered with the TB or app.

Preventive dental visit attendance of participating children will be ascertained through dental exam data from Head Start's ChildPlus Management Software health module. Access to and completeness of this data will be measured to assess feasibility of data collection.

4 STUDY DESIGN

This pilot study uses a 3-arm parallel design to compare the acceptability and feasibility of two micro-incentive modalities. We will use a stratified balance assignment to assign 75 child/caregiver dyads to three groups: Control, Fixed monetary incentives, and Drawing monetary incentives.

a. Pilot design and sample size. Child and parent/caregiver dyads meeting eligibility criteria will be randomly assigned to one of three groups: Control, Fixed payment incentives, or Drawing payment incentives (finalized based on UH2 Aim 1). The pilot trial sample size will be a total of 36 child-parent/caregiver dyads, with 12 in each group. (Sample size determination is explained in Section 12.2).

b. Micro-incentive payments. During the initial study visit, child and parent/caregiver dyads will be randomly assigned to a monetary incentive group or the control group, and the program procedures explained. If assigned to an incentive group, two different monetary incentives can be earned and will be paid out separately, based upon two criteria: (1) regularly brushing the participating child's teeth based on smart powered TB data and (2) among children lacking a baseline child dental exam documented in the Head Start ChildPlus database, a child dental exam documented in ChildPlus by the 2-month study follow-up visit. Toothbrushing performance will be assessed at one-week intervals. Caregivers in the drawing payment group will be informed that the drawing will occur every week throughout the 2-month pilot, and caregivers in the fixed payment group will be informed that incentives will be earned every week. All study participants will be sent weekly SMS text messages with preventive behavior reminders, toothbrushing adherence status per smart powered TB data and incentives earned if assigned to an incentive group. All rewards will be claimed at the 2-month study visit. The control group will have the ability to earn the same expected maximum rewards in a delayed (waitlist enrolled) 2-month fixed reward program, but this will not be part of the formal study evaluation.

c. Pilot evaluation. As a general overview of activities: each participant will attend two in-person visits (baseline, 2 months) at one of three EHS sites. Each in-person visit will take approximately 2 hours. Bilingual-bicultural staff will conduct computer assisted personal interviews (CAPI) to administer fully structured, objective questionnaires and enter information directly into the secure, web-based data management system, Qualtrics. Dental examination screening data will be entered directly into REDCap in an eCRF adapted from the ASTDD Basic Screening Survey. The smart powered TB and TP pump will be provided at the baseline visit, and study staff will instruct parents/caregivers on their use. Staff will also provide fluoride TP tubes to families to reduce the possibility of other family members using the TP pump. Participants will be asked to bring the TP pump with them to the 2-month follow-up visit; staff will weigh the pump to ascertain this toothbrushing adherence measure. Depending on the information gathered from the qualitative phase of this UH2 study, parents/caregivers will be instructed to regularly (e.g.,

twice a week) download (sync) data from the smart powered TB to their phone app which then is uploaded to the manufacturer's web-based database (cloud) regarding the length of time the TB was used on the child.

Subject participation for this pilot study will take approximately 2-3 months to complete.

If more than one child in the household is eligible to participate in the study, the oldest eligible child will be the index child. The parent/caregiver will be instructed to have each study-eligible child use his/her own smart TB for the duration of the study, but only the index child's TB will be synced to the smart phone app. Each child participant's TB will have a label with his/her name put on the handle along with customized individual stickers to avoid confusion. Participants will be asked not to allow other people to use the study child's smart TB for the duration of the study.

5 STUDY ENROLLMENT AND WITHDRAWAL

5.1 Subject Inclusion Criteria

To be eligible to participate in this study, a parent/caregiver must meet all of the following criteria:

- Provide signed and dated informed consent form in English or Spanish.
- Agree to comply with all study procedures and be available for the duration of the study visit.
- Male or female, aged 18 and older.
- Speak, read, and write either English or Spanish.
- Be a parent or caregiver of a child at least 6 months old but less than 3.5 years (42 months), with at least 2 fully erupted teeth and enrolled in, or waitlisted for, one of the three participating Los Angeles County EHS home visit programs.
- Not be planning to move residence for the next 6 months outside the greater Los Angeles area.
- Own a smartphone with the Google Play or iTunes store and be willing to download the smart powered TB app
- Be willing to be contacted via text-messaging (SMS) for study related notifications, such as incentives earned or reminders to sync the TB

5.2 Subject Exclusion Criteria

A child who meets any of the following criteria will be excluded from participation in this study:

- Known allergic reaction to components of the study product(s).
- Uncooperative or behaviorally unsuited (assessed during a TB prophylaxis at the initial baseline study visit).
- A sibling of a child already enrolled in the study (the family's oldest child in the eligible age range will be the study child).
- Enrolled in foster care.
- Anything else that would place him/her at increased health risk or preclude the individual's full compliance with or completion of the study.

5.3 Strategies for Recruitment and Retention

Using the standardized Recruitment Script, research study personnel will contact EHS home visit program families to determine interest in study participation. This outreach may

occur at the EHS center, during EHS monthly parent meetings, or via telephone calls to interested parents/caregivers who contacted study staff.

Mailings and flyers with study recruitment information may also be sent out or posted in the EHS facilities. The monthly EHS newsletter may also include an invitation to participate in the pilot.

EHS families who have participated in previous BEECON surveys or interviews may also be contacted by study personnel to inquire about their interest in participating in the pilot.

5.4 Treatment Assignment Procedures

Because study participant retention is essential, research staff may attempt to contact participating parents/caregivers via postcard mailings and phone calls between the two visits throughout the study to update contact information, in addition to reminding them of the follow-up appointment. Additionally, the parents/caregivers may receive text reminders to sync the smart powered TB to their smartphone app and/or messages about the importance of an early dental visit, serving as other reminders of the project. Finally, all study participants will be sent weekly SMS text messages with preventive behavior reminders, twice daily toothbrushing adherence status per smart TB data, and the incentive earned if assigned to an incentive group.

Each participant will receive a $30 gift card at the end of the baseline visit, and another $30 gift card at the end of the 2-month follow-up visit as compensation for their time. Participants will also be eligible to receive additional gift cards for syncing the smart powered TB app data throughout the study period, paid at the 2-month follow-up visit. To encourage participants to bring the TP pump for weighing with them to the 2-month follow-up visit, they will be informed that they will be given the gift cards, a new TP pump, and replacement brush heads only if they return the TP pump.

5.5 Treatment Assignment Procedures

Based on multiple discussions with EHS staff, a trial with different programs will need to demonstrate fairness in a transparent way to the parents/caregivers. Thus, during the explanation of the study to the participant, randomization and the rewards program will be explained with a teachback method. Stratified permuted block randomization will be used to assign participating dyads to a study arm, stratifying on EHS site and varying block size to reduce probability of guessing the next assignment. Once eligibility is confirmed and informed consent is documented, the project coordinator will open a sealed opaque envelope with the participant’s study ID number to reveal group assignment for the parent/caregiver-child dyad.

5.5.1 *Masking Procedures (if applicable)*

Independent dental examiners, PIs, and EHS home visitor staff (one per EHS site) will be blinded to participants’ group assignment and participants will be told those staff members

cannot answer any incentive questions and they should not mention the incentive program to them. The researcher providing the incentive gift cards will not be blinded. Dental screenings will precede monetary incentive payment.

5.6 Subject Withdrawal

Participants may withdraw voluntarily from the study at any time.

5.6.1 *Reasons for Withdrawal*

Participants may decide not to answer any CAPI item or not to participate in the study further at any time without penalty.

Participants are free to withdraw from participation in the study at any time upon request, for any reason, without penalty or loss of benefits as a member of the EHS program. This may occur due to discomfort with questions or practical concerns that limit the time available (e.g., pressing employment schedule or child care needs).

An investigator may terminate a study participant's participation in the study if:

- Any clinical adverse event (AE) or other medical condition or situation occurs such that continued participation in the study would not be in his/her best interest.
- The participant meets an exclusion criterion (either newly developed or not previously recognized) that precludes further study participation.

5.6.2 *Handling of Subject Withdrawals or Subject Discontinuation of Study Intervention*

If parent/caregiver informs the study staff that he/she does not wish to continue with the study any further, the staff member will document the reason(s) and remove the parent and child from the study. The participant will be encouraged to continue with regular dental examinations at the child's dental home, which could detect early signs of disease. A participant's withdrawal from this study will not influence his/her future relations with any of the study's collaborating institutions, including but not limited to LA County EHS, UCSF, UCLA, or the child's medical or dental care provider. Withdrawals will not be replaced. All study data provided before withdrawing will be used for analysis, unless the participant asks that her/his data and her/his child's data not be used.

5.7 Premature Termination or Suspension of Study

This study may be suspended or prematurely terminated if there is sufficient reasonable cause. Written notification, documenting the reason for study suspension or termination, will be provided by the suspending or terminating party to Drs. Stuart Gansky, James Kahn, and Francisco Ramos-Gomez (PIs). If the study is prematurely terminated or suspended, the PIs will promptly inform the IRB and will provide the reason(s) for the termination or suspension.

Circumstances that may warrant termination include, but are not limited to:

- Determination of unexpected, significant, or unacceptable risk to subjects.
- Insufficient adherence to protocol requirements.
- Data that are not sufficiently complete and/or evaluable.
- Determination of futility.

6 STUDY INTERVENTION

6.1 Description of Behavioral Intervention

Child-parent/caregiver dyads will be assigned in a 1:1:1 ratio to one of three conditions: Control group, a Fixed monetary incentive condition, or a Drawing monetary incentive condition.

In the Control group, participants will not be eligible to receive any performance-based incentives during the first 2 months, but will receive information on their toothbrushing adherence per the smart powered TB data, as well as other informational reminders about oral health care behaviors. After the 2-month follow-up visit, the control group will have the ability to earn Fixed incentives in a delayed (waitlisted) 2-month open label extension. This is not a formal part of the UH2 study evaluation for the future trial preparation, but rather a necessary condition of the trial design to assure that all participating EHS parents/caregivers have the chance to earn the same monetary incentives. They will follow the same procedures (texting notifications) as the Fixed intervention group from the 2-month follow-up to a 4-month follow-up. At the 4-month follow-up visit, they will be able to collect the earned incentives. To receive the rewards the parent/caregiver and child must attend the 4-month visit and return their toothpaste pump. Participants will be provided a new TP pump and 1 new brush head.

In the Fixed monetary incentive group, participants will be eligible to earn one of two incentive amounts every week. They will earn $5 in incentives if the participant meets a lower adherence threshold (i.e., likely 7 times brushing his/her child's teeth) during the preceding week. They will earn $10 in rewards if the participant meets a higher adherence threshold (i.e., likely 14 times brushing his/her child's teeth) during the preceding week. The exact thresholds may be modified based on UH2 formative research information being collected prior to the trial.

In the Drawing monetary incentive group, participants will be sent a text message about entering into a drawing every week; the participant can reply to the text with a 2-digit number or let his or her number be chosen at random. Chances of the amount of winnings possible depend on the participant's level of adherence. A participant who meets the lower adherence threshold (i.e., same as in the Fixed monetary incentive group; e.g. 7/week) will be eligible for an 18% chance (matching one digit with the winning number) of winning $25 and a 1% chance (matching both digits in order) of winning $50 (an expected value of $5). A participant who meets the higher adherence threshold (i.e., same as in the Fixed monetary incentive group; e.g. 14/week) will be eligible for a 34% chance (matching either digit without regard to order) of winning $25 and a 4% chance (matching both digits without regard to order) of winning $50 (an expected value of $10). Participants who fail to reach either adherence threshold can send a reply text message to enter into the lower adherence drawing, and if chosen as a winner will receive a text message that states what

they would have won had they brushed more regularly, thereby taking advantage of a psychological tendency toward anticipated regret to motivate future brushing.

6.2 Administration of Behavioral Intervention

All participants will be instructed to sync their smart powered TB data to the app twice per week. Failure to do so will disqualify the participant from receiving any incentives that week for those in an incentive group. All participants, regardless of group assignment, will receive a fixed payment of $3 per week for syncing their data every three days (Mondays, Wednesdays, and Fridays specifically). A text message reminder to sync the app will be sent to participants up to every 3 days. For those assigned to the Drawing incentive group, the reminder will also prompt participants to select a drawing number for the coming week. The drawing for performance-based incentives will take place about 72 hours after week's end.

First, all participants will enter a "practice" week at the start of the intervention, during which participants will sync their TB data (and be eligible for the $3 incentive for syncing), but not be eligible for the performance-based toothbrushing incentive. Following the practice week, participants are eligible to earn incentives over 8 one-week periods for a total of 56 days.

All participants will be notified by text message of their toothbrushing adherence status per the smart powered TB data every week. For participants assigned to a Fixed or Drawing incentive group, the text message will include the amount earned and the total balance accrued thus far during the 2-month pilot trial (i.e., gift cards, virtual drawing tickets and winnings, depending on parental/caregiver reward preferences obtained in the UH2 formative research). Participants will have the option to additionally have the text messages sent to other family members who regularly brush the child's teeth. Contact information for all family members will be obtained at the baseline visit and will initially require that the recipient opt-in: an introductory text message will be sent to the family members asking if they agree to receive the text messages and only those responding in the affirmative will receive text messages during the intervention.

Among children who did not have a documented ChildPlus dental visit at baseline, parents/caregivers in the Fixed incentive group will be eligible for a $40 reward if they take their child to a dental check-up visit and it is documented in the ChildPlus database by the 2-month follow-up visit. Among children without a ChildPlus documented baseline dental visit in the Drawing incentive group, parents/caregivers whose child has a documented ChildPlus dental visit by the 2-month follow-up will have a 10% chance of winning $400 in a drawing.

The monetary incentive groups will be told that having a preventive dental visit will earn them an incentive equivalent to 4 weeks of toothbrushing. Thus, the total expected value

of exceeding the higher-adherence threshold throughout the intervention period is $80 from toothbrushing and $40 from dental visit attendance.

To claim any incentives, including for syncing data, a parent/caregiver and child must attend the 2-month follow-up visit. All payments will be awarded as gift cards at the 2-month visit, but 2-month follow-up visit questionnaires will determine if another modality would be preferred in the future Phase II/III stage. After returning the TP pump participants will be provided a new TP pump and two new brush heads at the end of the intervention.

6.3 Procedures for Training Interventionists and Monitoring Intervention Fidelity

The Program Manager will train all research staff members on how to explain intervention procedures and incentive program details to participants. The Program Manager or another supervisor will assess fidelity from 100% of audio recordings during the training period and then from a randomly selected 20% of recordings thereafter every week during the expected 12-week participant accrual. A study-specific checklist of intervention procedures and incentive program details will be used to evaluate fidelity from the recordings. Checklists with less than 90% fidelity during the training period or during baseline visits will be declared inadequate and require re-training staff by the Program Manager. A study co-investigator will train the Program Manager to code fidelity data from the recordings.

6.4 Assessment of Subject Compliance with Study Intervention

Study team presentations to parents/caregivers will involve graphics and teachback to provide information about the randomization process and Fixed monetary incentive and Drawing monetary incentive packages. Presentations will be standardized to ensure consistency. Study team staff will document that the information was presented and that the parent/caregiver explained the procedures back in his/her own words. The parent/caregiver will sign to confirm he/she received the information. Throughout the study intervention, a random selection of 5% of SMS text messages to parents/caregivers will ask for a reply to verify receipt.

7 STUDY SCHEDULE

7.1 Screening

Study personnel will contact EHS families to assess eligibility and level of interest in participating in this study.

7.2 Enrollment/Baseline

Based on parent/caregivers' and participating EHS sites' availability, each child will be scheduled for the baseline visit with his/her parent/caregiver. When the family arrives at the EHS site, study personnel will explain in detail the goals and procedures of this pilot study.

Enrollment/Baseline Visit (Visit 1, T = 0)

- Explain purpose and procedures of study to prospective parent/caregiver participant. Verify inclusion/exclusion criteria.
- Obtain and document consent from subject on study consent form.
- Administer CAPI questionnaire to parent/caregiver.
- Perform dental screening on child participant's teeth (by blinded dental provider); record decay and assess treatment urgency. Children with urgent treatment needs will be referred with a warm handoff to staff at the corresponding dental clinic.
- Apply GUM Red-Cote Liquid plaque disclosing agent to child's teeth (by blinded dental provider) and record plaque score.
- Take extraoral photographs of the facial surfaces of child's anterior teeth.
- Give the family the Oral Hygiene Kit, which includes the smart powered TB and fluoride TP pump. Attach label with child's name.
- Instruct the parent/caregiver on how to sync the TB to his/her smartphone, use the TP pump to show the proper amount of TP, and how to brush the child's teeth. Have the parent brush off the child's plaque disclosing solution.
- Apply 3M Vanish (fluoride varnish) to the child's teeth (by dental provider).
- Give parent/caregiver the opaque envelope with the child's sequence number on the outside (by unblinded study staff); parent/caregiver opens the envelope to reveal the randomized assignment to arm. Explain the intervention procedures and rewards program (if assigned to a reward arm) to the parent/caregiver and have her/him explain it back in her/his own words. Explain control procedures to the parent/caregiver and have her/him explain it back in her/his own words.
- Have the parent/caregiver watch the Anticipatory Guidance presentation on a tablet.

- Schedule the family's next visit (in approximately 3 months) and give the parent/caregiver $30 compensation for attending the baseline visit.

7.3 2-month Follow-Up Study Visit (Visit 2, T = day 60 ± 28)

The final study visit for participants randomized to the incentive groups is 2 months after the baseline visit. During this exit visit, a trained staff member will complete the final questionnaire and post-test with the parent/caregiver following the established protocol. The dental provider will perform an oral examination and will provide a referral to a dental home if one has not already been established.

- Administer questionnaire to parent/caregiver.
- Collect the parent/caregiver's child toothbrushing diary and TP pump. Record weight of TP pump.
- Perform dental screening on child participant's teeth (by blinded dental provider); record decay and assess treatment urgency. Children with urgent treatment needs will be referred with a warm handoff to staff at the corresponding dental clinic.
- Apply GUM Red-Cote Liquid plaque disclosing agent to child's teeth (by blinded dental provider) and record plaque score.
- Take extraoral photographs of the facial surfaces of child's anterior teeth.
- Watch the parent/caregiver brush the child's teeth and have the parent brush off the child's plaque disclosing solution.
- Give the parent/caregiver an electronic tablet to watch the Anticipatory Guidance presentation.
- Give the parent/caregiver the accumulated rewards per assigned study group arm. Distribute new TB heads (2 TB heads for the intervention groups; 2 TB heads for control group participants who do not want to continue in the open label Fixed incentives for 2 months; 1 TB head for the control group participants who continue in the open label Fixed incentives program for 2months), TP pump.
- Give the parent/caregiver $30 compensation for attending the follow-up visit.
- Optional for Control Group only: Explain the intervention procedures and fixed rewards program to the parent/caregiver and have her/him explain it back in her/his own words (teachback).

7.4 4-month Follow-up Study Visit (Optional for Control Group Only, Visit 3, T = 120 ± 28)

The final study visit for participants randomized to the delayed control group is 2 months after the 2-month follow-up visit (i.e. 4-months from baseline). The dental provider will perform an oral examination and provide a referral to a dental home if one has not already been established.

- Collect the parent/caregiver's child toothbrushing diary and TP pump.
- Perform dental screening on child participant's teeth (by dental provider); record decay and assess treatment urgency. Children with urgent treatment needs will be referred with a warm handoff to staff at the corresponding dental clinic.
- Give the parent/caregiver an electronic tablet to watch the Anticipatory Guidance presentation.
- Give the parent/caregiver the accumulated fixed rewards. Distribute new brush head and a full TP pump.
- Give the parent/caregiver $30 compensation for attending the follow-up visit.

7.5 Withdrawal Visit

No early termination or withdrawal visits are anticipated, but in the event that this occurs, such a visit would follow the same guidelines established for a final study visit as long as the parent/caregiver is willing.

7.6 Unscheduled Visit

No unscheduled visits are anticipated. All adverse events will be reported as outlined in Section 9.

8 STUDY PROCEDURES / EVALUATIONS

8.1 Study Procedures/Evaluations

The clinical procedures and evaluations for this study will consist of the following: Child's medical history will be evaluated via a standardized questionnaire administered to the parent/caregiver at the dental screening visit. A dental provider will perform a dental screening examination and will obtain a plaque score on the buccal surfaces of anterior teeth after application of disclosing solution, following the Debris Index of the Oral Hygiene Index (Greene and Vermillion). Photographs of anterior teeth will be taken with the study camera to record plaque levels. After study staff demonstrate and observe parent/caregiver performance of study procedures, the dental provider will apply fluoride varnish.

8.2 Questionnaires

Research staff will administer CAPI Questionnaires to parents/caregivers during the study visits at baseline and 2-month follow-up, with data entered directly into Qualtrics; questionnaire data will be analyzed for changes in self-reported behaviors and perceptions, including relevant common data elements (CDEs) identified by NIDCR OHDC Collaborative Working Groups. Questionnaire items include demographics, current brushing behaviors, dental visit attendance drawn from BRFQ (Albino et al., in submission), economic module, and POQL (Huntington et al. 2011). Follow-up questionnaires will assess satisfaction/acceptance of the incentives and study procedures, as well as compliance with study procedures, such as whether others in the household used the child's powered toothbrush or toothpaste pump.

8.3 Anticipatory Guidance

A short health education video in English or Spanish will provide parents/caregivers with key preventive oral health messages appropriate for the child's age, such as the importance of fluoride supplements, healthy snacking and how to prevent the spread of bacteria that causes caries.

8.4 Toothbrushing Performance and Frequency

8.4.1 *Toothbrushing Diaries*

Parents/caregivers will be given calendar diaries to track toothbrushing events that can be compared to the smart powered TB's data. The calendar will be explained at baseline by research staff and collected at follow-up.

8.4.2 *Smart Toothbrushes*

During the baseline visit, all participants will receive a "smart" powered TB (e.g. Philips Oral Healthcare). The TB uses Bluetooth technology to transmit data on brushing timing,

frequency, and duration from the TB to an app installed on the participant's smartphone. Every week, the vendor (e.g. Philips Oral Healthcare) will transmit or provide a secure login to access an encrypted, de-identified file containing participants' brushing data to study investigators.

8.5 Dental Visit Attendance

Preventive dental visit attendance of participating children will be ascertained through dental exam data from Head Start's ChildPlus Management Software health module. In the subset of children at baseline without any dental visit in the Early Head Start ChildPlus health module, whether or not the child has a ChildPlus documented dental visit at the two-month follow-up will be assessed.

8.6 Study Products

The Program Manager will maintain a log of disclosing solution and TP shipments, including batch numbers and expiration dates. The study products will be stored in the Program Manager's office at UCLA.

8.6.1 *Plaque Disclosing Solution*

At each in-person visit, the dental provider will apply GUM Red-Cote Liquid plaque disclosing agent to the child's teeth for plaque assessment.

8.6.2 *Fluoride Varnish*

Vanish Fluoride Varnish (3M) is a varnish that is applied to the teeth and enhances enamel acid and boosts salivary fluoride levels. The product contains fluoride, calcium and phosphate, which helps the product remain on the teeth longer and provides extra protection. Vanish can be applied quickly without color change to the teeth. A dental clinician will apply the disclosing solution with a brush.

8.6.3 *Fluoride Toothpaste Pump*

Study participants will be instructed to use fluoridated TP based on the recent ADA consensus guideline (2014) that recommends all children use fluoride TP to help prevent dental disease. The study will use Colgate Maximum Cavity Protection Pump Fluoride Toothpaste (Colgate-Palmolive Company) (mild bubble fruit flavored).

The parents/caregivers will be instructed on how to dispense a smear or pea-sized amount (depending on child's age) of fluoride TP onto the TB for the child, as per study guidelines.

If a child experiences an adverse reaction to the fluoride TP, he/she will be provided clinical guidance (e.g., discontinue use of the product); based on clinical judgment and appropriate input from oversight groups, use may be re-started. However, no modification of study product dosage will be made.

9 ASSESSMENT OF SAFETY

9.1 Specification of Safety Parameters

The principal investigator (PI) will report safety events for the study (unanticipated problems [UPs], adverse events [AEs], serious adverse events [SAEs]) to the Institutional Review Board (IRB) in accordance with the IRB's requirements.

The PI will also report UPs involving risks to subjects to NIDCR. This will include UPs that meet the definition of a SAE. AEs that are temporally associated with the administration by study personnel of fluoride varnish will be recorded in an adverse event log, and this log will be monitored to identify events that meet the definition of a UP. If a UP is identified, it must be reported.

9.1.1 *Unanticipated Problems (UPs)*

The Office for Human Research Protections (OHRP) considers unanticipated problems (UPs) involving risks to subjects or others to include, in general, any incident, experience, or outcome that meets **all** of the following criteria:

- Unexpected in terms of nature, severity, or frequency given (a) the research procedures that are described in the protocol-related documents, such as the IRB-approved research protocol and informed consent document; and (b) the characteristics of the subject population being studied;
- Related or possibly related to participation in the research ("possibly related" means there is a reasonable possibility that the incident, experience, or outcome may have been caused by the procedures involved in the research); and
- Suggests that the research places subjects or others at a greater risk of harm (including physical, psychological, economic, or social harm) than was previously known or recognized.

9.1.2 *Adverse Events*

OHRP defines an adverse event as any untoward or unfavorable medical occurrence in a human subject, including any abnormal sign (for example, abnormal physical exam or laboratory finding), symptom, or disease, temporally associated with the subject's participation in the research, whether or not considered related to the subject's participation in the research (modified from the definition of adverse events in the 1996 International Conference on Harmonization E-6 Guidelines for Good Clinical Practice).

In this study, due to the anticipated low risk of products being used, AEs that are not acute with onset within three days of product administration or were not medically attended events, serious AEs (SAEs) or unanticipated problems related to the intervention (UPs) will not be collected.

SAEs and UPs will be captured throughout the study period.

All reportable AEs will be graded for severity and relationship to the study product (see Section 9.3.3).

9.1.3 *Serious Adverse Events (SAEs)*

A serious adverse event (SAE) is one that meets one or more of the following criteria:

- Results in death.
- Is life-threatening (places the subject at immediate risk of death from the event as it occurred).
- Results in inpatient hospitalization or prolongation of existing hospitalization.
- Results in a persistent or significant disability or incapacity.
- Results in a congenital anomaly or birth defect.
- An important medical event that may not result in death, be life threatening, or require hospitalization may be considered an SAE when, based upon appropriate medical judgment, the event may jeopardize the subject and may require medical or surgical intervention to prevent one of the outcomes listed in this definition.

9.2 Time Period and Frequency for Event Assessment and Follow-Up

AEs, UPs and SAEs will be recorded in the data collection system throughout the study. Events will be followed for outcome information until resolution or stabilization.

The Study PI will record all reportable events with start dates occurring any time after informed consent is obtained until seven (for non-serious AEs) or 30 days (for SAEs) after the last day of study participation. Parents/caregivers will be instructed to report any doctor office or emergency room visits in the three days after the fluoride varnish application. At the 2-month follow-up (final) study visit, the investigator will inquire about the occurrence of AE/SAEs including FV-related AE/SAEs since the last visit. Events will be followed for outcome information until resolution or stabilization.

9.3 Characteristics of an Adverse Event

9.3.1 *Relationship to Study Intervention*

To assess relationship of an event to study intervention, the following guidelines are used:

1. Related (Possible, Probable, Definite)
    a. The event is known to occur with the study intervention.

   b. There is a temporal relationship between the intervention and event onset.
   c. The event abates when the intervention is discontinued.
   d. The event reappears upon a re-challenge with the intervention.
2. Not Related (Unlikely, Not Related)
   a. There is no temporal relationship between the intervention and event onset.
   b. An alternate etiology has been established.

9.3.2 *Expectedness of AEs*

The Study PI will be responsible for determining whether a SAE is expected or unexpected. An AE will be considered unexpected if the nature, severity, or frequency of the event is not consistent with the risk information previously described for the intervention.

9.3.3 *Severity of Event*

Severity of Event: All reportable AEs will be assessed by a clinician study PI or co-investigator using the following guidelines to quantify intensity:

1) Mild: events require minimal or no treatment and do not interfere with the participant's daily activities.

2) Moderate: events result in a low level of inconvenience or concern with the therapeutic measures. Moderate events may cause some interference with functioning.

3) Severe: events interrupt a participant's usual daily activity and may require systemic drug therapy or other treatment. Severe events are usually incapacitating

9.4 Reporting Procedures

9.4.1 *Unanticipated Problem (UP) Reporting to IRB and NIDCR*

Incidents or events that meet the OHRP criteria for UPs require the creation and completion of an UP report form. OHRP recommends that PIs include the following information when reporting an AE, or any other incident, experience, or outcome as an UP to the IRB:

- Appropriate identifying information for the research protocol, such as the title, investigator's name, and the IRB project number;
- A detailed description of the AE, incident, experience, or outcome;

- An explanation of the basis for determining that the AE, incident, experience, or outcome represents an UP;
- A description of any changes to the protocol or other corrective actions that have been taken or are proposed in response to the UP.

To satisfy the requirement for prompt reporting, UPs will be reported using the following timeline:

- UPs that are SAEs will be reported to the UCSF IRB (CHR) and to NIDCR within 5 work days of a PI becoming aware of the event.
- Any other UP will be reported to the IRB and to NIDCR within 10 work days of a PI becoming aware of the problem.
- All UPs should be reported to appropriate institutional officials (as required by an institution's written reporting procedures), the supporting agency head (or designee), and OHRP within one (1) month of the IRB's receipt of the report of the problem from a PI.

All UPs will be reported to NIDCR's centralized reporting system via Rho Product Safety:

- Product Safety Fax Line (US): 1-888-746-3293
- Product Safety Fax Line (International): 1-919-287-3998
- Product Safety Email: *rho_productsafety@rhoworld.com*
- CC Safety Email: *OHDCSAE@ucsf.edu*
- CC Safety Fax Line: 1-415-502-8447

General questions about SAE reporting can be directed to the Rho Product Safety Help Line (available 8:00AM – 5:00PM Eastern Time):

- US: 1-888-746-7231
- International: 1-919-595-6486

9.4.2 *Serious Adverse Event Reporting to IRB and NIDCR*

In the unlikely event that an AE or SAE incident occurs during this pilot study, any AE meeting the specified SAE criteria will be submitted within 10 work days of a PI's awareness in the UCSF electronic IRB system to the UCSF CHR and on an SAE form to NIDCR's centralized safety system via Rho Product Safety. This report may be sent by fax or email. Once submitted, Rho Product Safety will send a confirmation email to the PIs within one (1) business day. The investigator should contact Rho Product Safety if this confirmation is not received. This process applies to both initial and follow-up SAE reports.

SAE Reporting Contact Information:

- Product Safety Fax Line (US): 1-888-746-3293
- Product Safety Fax Line (International): 919-287-3998
- Product Safety Email: *rho_productsafety@rhoworld.com*
- CC Safety Email: *OHDCSAE@ucsf.edu*
- CC Safety Fax Line: 1-415-502-8447

General questions about SAE reporting can be directed to the Rho Product Safety Help Line (available 8:00AM – 5:00PM Eastern Time):

- US: 1-888-746-7231
- International: 919-595-6486

The study clinician will complete a SAE form and submit via fax or email within the following timelines:

- All deaths and immediately life-threatening events, whether related or unrelated, will be recorded on the SAE form and submitted to Product Safety within 24 hours of site awareness.
- SAEs other than death and immediately life-threatening events, regardless of relationship, will be reported by fax within 5 work days of site awareness.

All SAEs will be followed until resolution or stabilization.

9.4.3 *Reporting of SAEs and AEs to FDA*

Any AE meeting the specified SAE criteria and being rated as related (possible, probable, definite) to fluoride varnish administration will be submitted to the FDA using the MedWatch voluntary reporting system (*https://www.accessdata.fda.gov/scripts/medwatch/index.cfm?action=reporting.home*).

9.4.4 *Events of Special Interest (if applicable)*

Suspected child abuse reporting is mandatory because the study participants include minors; any incidents will be reported to an EHS supervisor. California law stipulates that a provider seeing a child as a patient is not required to report domestic violence or abuse of the parent/caregiver. But the parent/caregiver may choose to make his/her own report by contacting police. We do not anticipate any other events of special interest during the short duration of this study.

9.5 Halting Rules

The frequency of AEs meeting criteria defined in Section 9.3.1 is expected to be very small. If the number of SAEs is large, or of a particular type, NIDCR may temporarily suspend enrollment until a safety review is convened. The objective of such a review

would be to decide whether the study should continue per protocol, proceed with caution, be further investigated, be modified and then continued, or be discontinued.

10 STUDY OVERSIGHT

The principal investigators will be responsible for study oversight, including monitoring safety, ensuring that the study is conducted according to the protocol and ensuring data integrity. The PIs will review the data for safety concerns and data trends at regular intervals, and will promptly report to the IRB and NIDCR any Unanticipated Problem (UP), protocol deviation, or any other significant event that arises during the conduct of the study.

11 CLINICAL SITE MONITORING

The principal investigators and the study team will conduct quality management activities as described in Section 14. There will be no external clinical site monitoring for this developmental pilot study. The PIs will provide monitoring in accordance with the IRB policies. The NIDCR reserves the right to conduct independent audits as necessary.

12 STATISTICAL CONSIDERATIONS

12.1 Study Hypotheses

Overall, we hypothesize that one reward strategy informed by behavioral economic theory will be more promising for promoting preventive dental care behaviors by parents/caregivers for their children. The pilot trial hypotheses are:

(a) At least one monetary reward arm (Fixed or Drawing) will have at least 50% of potential participants hypothetically agree to participate and at least 80% of participants willing to accept randomization to an incentive arm (including non-monetary reward control).

(b) The remaining study objectives are hypothesis-generating and enabling precise sample size calculations for the future Phase II/III trial.

12.2 Sample Size Considerations

The pilot trial sample size will not predominantly be driven by statistical power for group comparisons, but rather the sample size to provide estimates and reasonably precise (narrow) confidence intervals (CIs) of effect sizes to be used to plan the future Phase II/III trial. For example, with n=12 per group and alpha=0.05, the 95% CI for a proportion is estimated to extend 0.100 to 0.283 from the observed proportion depending on the underlying proportion (ranging from 0.032 to 0.968 with 0.5 having the widest interval). Nevertheless, with 3 groups of 12 and alpha=0.05: (1) a one-way analysis of variance is estimated to have 80% power to detect a difference in percent change means of feasibility / acceptability measures when variance of means is 0.024 and a common standard deviation is 0.286 (smaller standard deviations will have greater power); (2) a chi-square test of feasibility / acceptability measures is estimated to have 80% power to detect a difference in proportions characterized when the variance of proportions of 0.066 and an average proportion of 0.547.

12.3 Final Analysis Plan

Two types of data analysis will be conducted for the pilot UH2:

(1) <u>Quantitative analysis:</u> To refine sample size, regression analyses (adjusted for *a priori* potential confounders) will be used to estimate differences and confidence intervals among the three arms between baseline 2-month follow-up visits for the percent change in (1) toothbrushing with fluoridated TP, (2) ChildPlus documented dental visits, and (3) both outcomes combined. Percentages and 95% CIs will be estimated for all feasibility (willingness) measures.

(2) <u>Qualitative exploratory analysis:</u> Perceptions of incentives, their conditionality and achievability, and value of proper oral health care for children will be compared by sociodemographic characteristics. We will select the reward arm where at least 50% of potential participants hypothetically agree to participate and in which at least 80% of

participants accept randomization to a reward arm (including the non-monetary reward control). These will be determined at the 2-month follow-up visit with structured and open-ended questionnaires regarding pilot participation.

Among groups declared “acceptable”, “feasible” and “appropriate”, the group with outcomes at least 20% better than the other two groups will be chosen. If no group is at least 20% better, then the one that either 1) has the highest adherence or 2) is declared most “appropriate” (e.g. easier to explain and understand or less resource intensive) will be chosen for the future Phase II/III trial.

Descriptive statistics (means, standard deviations, frequencies, 95% confidence intervals) will be used to summarize the group specific findings. An intention-to-treat (as assigned) approach will be used. Group estimates and percent difference for parents/caregivers brushing children’s teeth, ChildPlus documented dental attendance, EHS staff participation, parent/caregiver participation (informed consent), willingness to be randomized, willingness to sync smart TB to app, acceptability of the smart powered TB, child willingness to cooperate with dental screening, extraoral photo and plaque assessment, willingness to bring the TP pump to the 2-month visit, item completion/missingness among the 3 groups will be estimated with standard errors and 95% confidence intervals (CIs) to provide valid and precise estimates for sample size calculations for the future Phase II/III trial. If data are not Normally distributed, Normalizing transformations or nonparametric methods will be used. Multiple imputation models will be used for missing data. Although this pilot is not primarily focused on testing for statistically significant differences among groups, analysis of covariance (or nonparametric analysis of covariance) adjusting for potential confounders will be used to estimate effect sizes of the two monetary reward groups versus the control non-monetary reward condition to be used in sample size calculations.

Toothbrushing measures (smart powered TB, parent/caregiver diaries), child plaque scores, TP usage (change in pump weight) will be compared with Pearson (or nonparametric Spearman) correlation coefficients to assess consistency in the various measures. Toothbrushing measures (smart poweredTB, parent/caregiver diaries) will be examined overall and by group over time (2 months) to assess for possible temporal effects (e.g., short-term smart powered TB novelty effect). Validity of the TB measures will be assessed by evaluating concordance of: TB diaries with smartphone app TB data; TB diaries with DI plaque score; TB diaries with TP pump weight change; smartphone app TB data with DI plaque score; smartphone app TB data with TP pump weight change; and DI plaque score with TP pump weight change.

13 SOURCE DOCUMENTS AND ACCESS TO SOURCE DATA/DOCUMENTS

Study staff will maintain appropriate medical and research records for this study, in compliance with ICH E6, Section 4.9 and regulatory and institutional requirements for the protection of confidentiality of subjects. Study staff will permit authorized representatives of NIDCR and regulatory agencies to examine (and when required by applicable law, to copy) research records for the purposes of quality assurance reviews, audits, and evaluation of the study safety, progress and data validity.

Data collected in this study will take the form of parent/caregiver questionnaires administered in structured CAPIs and dental examination data. Source documents for this study are: parent/caregiver questionnaires collected with electronic case report forms (eCRFs) during structured interviews, paper parent/caregiver TB diaries, electronic smart TB performance data, and dental examination data which will be collected electronically in REDCap. All data and source documents will be stored on password-protected computers with full hard drive encryption and secure servers behind a firewall. Paper informed consent forms will be stored in locked cabinets.

14 QUALITY CONTROL AND QUALITY ASSURANCE

All staff will be appropriately trained in his/her position's duties with appropriate Human Subjects Research; they will be provided with full information about the study such as background and significance, specific aims, milestones, study designs, procedures and analytic plans. All team members will be encouraged to view themselves as crucial team members encouraged to comment, ask questions, and make suggestions to ensure the highest study quality; robust and rigorous data will be gathered and handled according to protocol.

Working with the NIDCR designated U01 Coordinating Center (CC), we will use the REDCap web-based clinical trials management system (CTMS) to manage the trial visit schedule, group assignment, electronic case report forms (eCRFs), and AE and UP reporting. After training from the CC, staff will directly enter participant data into the CTMS.

Conventional data-checking routines will assess data completeness and will ensure data are input and output successfully and within reasonable ranges. Checks will be done shortly after data collection. After data-cleaning procedures, files for specific data collection will be validated and locked. Additionally reports of electronic data collected will be obtained and reviewed with the study team for quality management.

The study PIs will rigorously and periodically monitor study activities and engage in study team meetings to ensure that procedures are being performed correctly in the field in the proper sequence. If any breach is discovered, the particular activity will be undertaken a second time and monitored closely for full compliance with the approved procedures.

15 ETHICS/PROTECTION OF HUMAN SUBJECTS

15.1 Ethical Standard

The investigator will ensure that this study is conducted in full conformity with the principles set forth in The Belmont Report: Ethical Principles and Guidelines for the Protection of Human Subjects of Research, as drafted by the US National Commission for the Protection of Human Subjects of Biomedical and Behavioral Research (April 18, 1979) and codified in 45 CFR Part 46 and/or the ICH E6.

15.2 Institutional Review Board (IRB)

The protocol, informed consent form(s), recruitment materials, and all subject materials will be submitted to the IRB for review and approval. Approval of both the protocol and the consent form must be obtained before any subject is enrolled. Any amendment to the protocol, study procedures or instruments will require review and approval by the IRB before the changes are implemented in the study. UCSF's Committee on Human Research (CHR) will be the reviewing IRB while UCLA's CHR will be the relying IRB, formalized with an official Notice of Intent to Rely through the University of California system.

15.3 Informed Consent Process

Informed consent is a process that is initiated prior to the individual agreeing to participate in the study and continues throughout study participation. Discussion of risks and possible benefits of study participation will be provided to potential participants and their families, if applicable. A consent form describing in detail the study procedures and risks will be given to the participant. Consent forms will be IRB-approved, and the participant is required to read and review the document on a digital tablet in English or Spanish or have the document read to him or her. The investigator or designee will explain the research study to the participant and answer any questions that may arise. The participant will electronically sign (with UCSF IRB-approved DocuSign) the informed consent document prior to any study-related assessments or procedures. Participants will be given the opportunity to discuss the study with their surrogates or think about it prior to agreeing to participate. They may withdraw consent at any time throughout the course of the study. A copy of the electronic signed informed consent document will be emailed to participants along with the Bill of Experimental Subject's Rights in English or Spanish (as per California law) for their records; participants who do not have email will be provided a paper copy of each and/or PDF via SMS text. The rights and welfare of the subjects will be protected by emphasizing to them that the quality of their clinical care will not be adversely affected if they decline to participate in this study.

The consent process will be electronically documented in the clinical research record.

15.4 Exclusion of Women, Minorities, and Children (Special Populations)

Inclusion and exclusion criteria have been developed, which do not include any exclusions based on gender or race/ethnicity.

15.5 Subject Confidentiality

Subject confidentiality is strictly held in trust by the investigators, study staff, and the sponsor(s) and their agents.

The study protocol, documentation, data, and all other information generated will be held in strict confidence. No information concerning the study or the data will be released to any unauthorized third party without prior written approval of the sponsor.

Authorized representatives of the sponsor may inspect all study documents and records required to be maintained by the investigator for the study subjects. The clinical study site will permit access to such records.

No individual identities will be used in any reports or publications resulting from this study. No identifying information will be transmitted from the clinical site or UCSF to NIDCR.

Electronic case report forms (eCRFs), which contain the participants' personal identifying information will be kept separately from other data.

16 DATA HANDLING AND RECORD KEEPING

The investigators are responsible for ensuring the accuracy, completeness, legibility, and timeliness of the data reported. All source documents will be completed in a neat, legible manner to ensure accurate interpretation of data. The investigators will maintain adequate case histories of study subjects, including accurate electronic case report forms (eCRFs), and source documentation.

16.1 Data Management Responsibilities

Data collection and accurate documentation are the responsibility of the study staff under the supervision of the PIs. All source documents must be reviewed by the study team and data entry staff, who will ensure that they are accurate and complete. UPs and AEs must be reviewed by the PIs or their designees.

16.2 Data Capture Methods

Data stored electronically will include informed consent documents. REDCap eCRFs will include oral health screening data, parent/caregiver reported toothbrushing diary data and toothpaste pump weights; Qualtrics eCRFs will be used for questionnaire data. ChildPlus dental attendance visit data will be extracted from the EHS computer database. In case that method is not possible, data will be entered into an REDCap eCRF. Smart toothbrush toothbrushing data and SMS text message data will be obtained as separate data bases from the toothbrush vendor and the SMS text messaging provider.

16.3 Schedule and Content of Reports

The study team will utilize monthly reports and figures to monitor enrollment and retention and will provide monthly enrollment/retention reports to NIDCR for review. Accrual and data completion will be tabulated at least fortnightly. The analytic data base will be cleaned and locked as described in the OHDC CC CDMP.

16.4 Study Records Retention

Study records will be maintained for at least three years after the date that the grant federal financial report (FFR) is submitted to the NIH.

The study will maintain participant records, including data from dental records; electronic informed consent records for minors will be retained based on the UC Counsel General's recommendation, which is that records be kept for seven years after a child reaches 18 years old (the age of majority), or until the child is 25. This could mean that participant records would be retained for up to 25 years after the end of the study, depending upon enrollment date in the study.

Individuals will be assigned a unique study identification (ID) code number to be used on all study instruments, documents or files. A coding key containing the basic information

about participants (e.g., names) and their assigned study ID numbers will be securely kept (password protected and encrypted) in the REDCap CTMS. All de-identified data will be kept indefinitely.

Master files will be stored on a UCSF secure server (e.g., via password protection and encryption) for possible later use in specific analyses, and all other copies erased from any portable disk, flash drive, or other media.

16.5 Protocol Deviations

A protocol deviation is any noncompliance with the clinical study protocol, good clinical practice, or Manual of Procedures requirements. The noncompliance may be on the part of the participant, the investigator, or study staff. As a result of deviations, study staff may develop corrective actions, which will be implemented promptly.

These practices are consistent with investigator and sponsor obligations in ICH E6:

- Compliance with Protocol, Sections 4.5.1, 4.5.2, 4.5.3, and 4.5.4.
- Quality Assurance and Quality Control, Section 5.1.1
- Noncompliance, Sections 5.20.1 and 5.20.2.

All deviations from the protocol must be addressed in study subject source documents and promptly reported to NIDCR and the local IRB, according to their requirements.

17 PUBLICATION/DATA SHARING POLICY

A copy of all materials submitted for publication must be submitted in advance to the NIH Project Officer. In addition, one copy of each publication resulting from work performed under an NIH grant-supported project must accompany the annual progress report submitted to the NIH awarding office. (See online at *http://grants.nih.gov/grants/policy/nihgps_2001/part_iia_5.htm#NoncompetingContinuationAwards*.)

17.1 Peer Review

Co-investigators and study staff will provide internal peer review. Moreover, peer review may be sought from investigator colleagues in the UCSF Center to Address Disparities in Children's Oral Health (CAN DO) for presentations, publications and protocols. CAN DO's review system, modified if necessary, oversees reviewing each abstract, manuscript or proposal being submitted.

As with all NIDCR-supported research, all materials submitted for publication, publications and reports resulting from activities supported by this award must acknowledge support from the National Institute of Dental and Craniofacial Research, NIH, specifically referencing the grant or cooperative agreement number.

An acknowledgment shall be made to the effect that:

"This publication was made possible by Cooperative Agreement UH2 DE025514 from the US DHHS, National Institute of Dental and Craniofacial Research" **or** "The project described was supported by Cooperative Agreement UH2 DE025514 from US DHHS, National Institute of Dental and Craniofacial Research" **and** "Its contents are solely the responsibility of the authors and do not necessarily represent the official views of the National Institute of Dental and Craniofacial Research or National Institutes for Health."

In the event that the recipient wishes to join with NIH in a simultaneous news release announcing the results of a project, the action should be coordinated with the awarding office.

17.2 Authorship

Investigators are strongly encouraged to decide on authorship of scientific documents prior to writing them. The principles set forth in the International Committee of Medical Journal Editors' (ICMJE) Uniform Requirements for Manuscripts Submitted to Biomedical Journals (the "Vancouver Rules") are to be followed. These standards can be reviewed online at *http://www.icmje.org*.

In the event of disagreement, an ad hoc Publication Committee appointed by the Executive Committee will provide resolution.

This study will comply with the *policy*, which ensures that the public has access to the published results of NIH-funded research. It requires scientists to submit final peer-reviewed journal manuscripts that arise from NIH funds to the digital archive PubMed Central upon acceptance for publication.

The ICMJE member journals have adopted a clinical trials registration policy as a condition for publication. The ICMJE defines a clinical trial as any research project that prospectively assigns human subjects to intervention or concurrent comparison or control groups to study the cause-and-effect relationship between a medical intervention and a health outcome. Medical interventions include drugs, surgical procedures, devices, behavioral treatments, process-of-care changes, and the like. Health outcomes include any biomedical or health-related measures obtained in patients or participants, including pharmacokinetic measures and adverse events. The ICMJE policy requires that all clinical trials be registered in a public trials registry such as *ClinicalTrials.gov*, which is sponsored by the National Library of Medicine. Other biomedical journals are considering adopting similar policies. For interventional clinical trials performed under NIDCR grants and cooperative agreements, it is the grantee's responsibility to register the trial in an acceptable registry, so the research results may be considered for publication in ICMJE member journals. The ICMJE does not review specific studies to determine whether registration is necessary; instead, the committee recommends that researchers who have questions about the need to register err on the side of registration or consult the editorial office of the journal in which they wish to publish.

U.S. Public Law 110-85 (Food and Drug Administration Amendments Act of 2007 or FDAAA), Title VIII, Section 801 mandates that a "responsible party" (i.e., the sponsor or designated principal investigator) register and report results of certain "applicable clinical trials:"

Trials of Drugs and Biologics: Controlled, clinical investigations, other than Phase I investigations, of a product subject to FDA regulation;

Trials of Devices: Controlled trials with health outcomes of a product subject to FDA regulation (other than small feasibility studies) and pediatric postmarket surveillance studies.

NIH grantees must take specific steps with NIH implementation of FDAAA.

18 LITERATURE REFERENCES

American Academy of Pediatrics: Section on Pediatric Dentistry. Oral Health Risk Assessment Timing and Establishment of the Dental Home. *Pediatrics.* 111: 1113-1116, **2003**.

ADA statement on early childhood caries. http://www.ada.org/en/about-the-ada/ada-positions-policies-and-statements/statement-on-early-childhood-caries (Jun. 18, 2014),

Banerjee AV, Duflo E, Glennerster R & Kothari D. Improving immunisation coverage in rural India: clustered randomised controlled evaluation of immunisation campaigns with and without incentives. *BMJ.* 340: c2220, **2010**. PMC2871989.

Bénabou R & Tirole J. Intrinsic and Extrinsic Motivation. *Review of Economic Studies.* 70: 489-520, **2003**.

Bénabou R & Tirole J. Incentives and Prosocial Behavior. *American Economic Review.* 96: 1652-1678, **2006**.

Birkhead GS, LeBaron CW, Parsons P, Grabau JC, Maes E, Barr-Gale L, Fuhrman J, Brooks S, Rosenthal J, Hadler SC & et al. The immunization of children enrolled in the Special Supplemental Food Program for Women, Infants, and Children (WIC). The impact of different strategies. *JAMA.* 274: 312-6, **1995**.

Burns T. A Structural Theory of Social Exchange. *Acta Sociologica.* 16: 188-208, **1973**.

Butani Y, Weintraub JA & Barker JC. Oral health-related cultural beliefs for four racial/ethnic groups: Assessment of the literature. *BMC Oral Health.* 8: 26, **2008**. PMC2566974.

Centers for Disease Control. *Promoting oral health: interventions for preventing dental cares, oral and pharyngeal cancers, and sports-related craniofacial injuries. A report on recommendations of the task force on community preventive services.* Centers for Disease Control: 2001; pp 1-13.

Creeth JE, Gallagher A, Sowinski J, Bowman J, Barrett K, Lowe S, Patel K, Bosma ML. The effect of brushing time and dentifrice on dental plaque removal in vivo. *J Dent Hyg*. 83(3):111-6. **2009**

Curry SJ, Wagner EH & Grothaus LC. Evaluation of intrinsic and extrinsic motivation interventions with a self-help smoking cessation program. *J Consult Clin Psychol.* 59: 318-24, **1991**.

Das J, Do QT & Ozler B. Reassessing conditional cash transfer programs. *World Bank Research Observer.* 20: 57-80, **2005**.

Della Vigna S & Malmendier U. Paying Not to Go to the Gym. *American Economic Review.* 96: 694-719, **2006**.

Dental Health Foundation. *“Mommy, It Hurts to Chew” The California Smile Survey: An Oral Health Assessment of California’s Kindergarten and 3rd Grade Children*; Dental Health Foundation: 2006.

Deren S, Stephens R, Davis WR, Feucht TE & Tortu S. The impact of providing incentives for attendance at AIDS prevention sessions. *Public Health Rep.* 109: 548-54, **1994**. PMC1403532.

Dey P, Foy R, Woodman M, Fullard B & Gibbs A. Should smoking cessation cost a packet? A pilot randomized controlled trial of the cost-effectiveness of distributing nicotine therapy free of charge. *Br J Gen Pract.* 49: 127-8, **1999**. PMC1313349.

Dye BA, Tan S, Smith V, Lewis BG, Barker LK, Thornton-Evans G, Eke PI, Beltran-Aguilar ED, Horowitz AM & Li CH. Trends in oral health status: United States, 1988-1994 and 1999-2004. *Vital Health Stat 11.* 1-92, **2007**.

Dye BA, Thornton-Evans G, Li X, Iafolla TJ. Dental caries and sealant prevalence in children and adolescents in the United States, 2011–2012. NCHS data brief, no 191. Hyattsville, MD: National Center for Health Statistics. 2015.Gansky SA, Ramos-Gomez F, Mertz E & Cheng N *Fluoride varnish can prevent caries in California's low-income preschool children*; California Policy Research Center, University of California, Office of the President: California Program on Access to Care Findings, 2007.

Finkelstein EA, Linnan LA, Tate DF & Birken BE. A pilot study testing the effect of different levels of financial incentives on weight loss among overweight employees. *J Occup Environ Med.* 49: 981-9, **2007**.

General Accounting Office (GAO). *DENTAL SERVICES: Information on Coverage, Payments, and Fee Variation*. GAO–13-754. Washington, DC: September 6, 2013. http://www.gao.gov/assets/660/657454.pdf

Gneezy U & Rustichini A. Pay enough or don't pay at all. *Quarterly Journal of Economics.* 115: 791-810, **2000**.

Goette LF & Stutzer A. Blood Donations and Incentives: Evidence from a Field Experiment. *IZA Discussion Papers.* No. 3580, **2008**. http://ssrn.com/abstract=1158977

Greenwell AL, Johnsen D, DiSantis TA, Gerstenmaier J & Limbert N. Longitudinal evaluation of caries patterns form the primary to the mixed dentition. *Pediatr Dent.* 12: 278-82, **1990**.

Grim CW, Broderick EB, Jasper B & Phipps KR. A comparison of dental caries experience in Native American and Caucasian children in Oklahoma. *J Public Health Dent.* 54: 220-7, **1994**.

John LK, Loewenstein G, Troxel AB, Norton L, Fassbender JE & Volpp KG. Financial incentives for extended weight loss: a randomized, controlled trial. *J Gen Intern Med.* 26: 621-6, **2011**. PMC3101962.

Haisley E, Volpp KG, Pellathy T & Loewenstein G. The impact of alternative incentive schemes on completion of health risk assessments. *Am J Health Promot.* 26: 184-8, **2012**.

Handa S & Davis B. The experience of conditional cash transfers in Latin America and the Caribbean. *Development Policy Review.* 24: 513-536, **2006**.

Herring P, Montgomery S, Yancey AK, Williams D & Fraser G. Understanding the challenges in recruiting blacks to a longitudinal cohort study: the Adventist health study. *Ethn Dis.* 14: 423-30, **2004**.

Homans GC, *Social behavior: Its elementary forms. (Revised ed.)*. Harcourt Brace Jovanovich: Oxford, England, **1974**; p xi, 386.

Huntington NL, Spetter D, Jones JA, Rich SE, Garcia RI, Avron S. Development and validation of a measure of pediatric oral health-related quality of life: the POQL. J Pub Health Dent. 71(3):185-193, **2011**.

Kamb ML, Rhodes F, Hoxworth T, Rogers J, Lentz A, Kent C, MacGowen R & Peterman TA. What about money? Effect of small monetary incentives on enrollment, retention, and motivation to change behaviour in an HIV/STD prevention counselling intervention. The Project RESPECT Study Group. *Sex Transm Infect.* 74: 253-5, **1998**. PMC1758123.

Kane RL, Johnson PE, Town RJ & Butler M. A structured review of the effect of economic incentives on consumers' preventive behavior. *Am J Prev Med.* 27: 327-52, **2004**.

Kaste LM, Marianos D, Chang R & Phipps KR. The assessment of nursing caries and its relationship to high caries in the permanent dentition. *J Public Health Dent.* 52: 64-8, **1992**.

Kranz AM, Rozier RG, Preisser JS, Stearns SC, Weinberger M & Lee JY. Comparing medical and dental providers of oral health services on early dental caries experience. *Am J Public Health.* 104: e92-9, **2014**. PMC4056232.

Lacetera N & Macis M. Do all material incentives for pro-social activities backfire? The response to cash and non-cash incentives for blood donations. *Journal of Economic Psychology.* 31: 738-748, **2010**.

Laken MP & Ager J. Using incentives to increase participation in prenatal care. *Obstet Gynecol.* 85: 326-9, **1995**.

Lee JY, Bouwens TJ, Savage MF & Vann WF, Jr. Examining the cost-effectiveness of early dental visits. *Pediatr Dent.* 28: 102-5; discussion 192-8, **2006**.

Lee SK & Cheng YY. Reaching Asian Americans: sampling strategies and incentives. *J Immigr Minor Health.* 8: 245-50, **2006**.

Lussier JP, Heil SH, Mongeon JA, Badger GJ & Higgins ST. A meta-analysis of voucher-based reinforcement therapy for substance use disorders. *Addiction.* 101: 192-203, **2006**.

Malotte CK, Hollingshead JR & Rhodes F. Monetary versus nonmonetary incentives for TB skin test reading among drug users. *Am J Prev Med.* 16: 182-8, **1999**.

Malotte CK, Rhodes F & Mais KE. Tuberculosis screening and compliance with return for skin test reading among active drug users. *Am J Public Health.* 88: 792-6, **1998**. PMC1508952.

Marcus AC, Crane LA, Kaplan CP, Reading AE, Savage E, Gunning J, Bernstein G & Berek JS. Improving adherence to screening follow-up among women with abnormal Pap smears: results from a large clinic-based trial of three intervention strategies. *Med Care.* 30: 216-30, **1992**.

Marteau TM, Ashcroft RE & Oliver A. Using financial incentives to achieve healthy behaviour. *BMJ.* 338: b1415, **2009**.

Martinez-Ebers V. Using Monetary Incentives with Hard-to-Reach Populations in Panel Surveys. *International Journal of Public Opinion Research.* 9: 77-86, **1997**.

Mayer JA & Kellogg MC. Promoting mammography appointment making. *J Behav Med.* 12: 605-11, **1989**.

Melnikow J, Paliescheskey M & Stewart GK. Effect of a transportation incentive on compliance with the first prenatal appointment: a randomized trial. *Obstet Gynecol.* 89: 1023-7, **1997**.

Nexoe J, Kragstrup J & Ronne T. Impact of postal invitations and user fee on influenza vaccination rates among the elderly. A randomized controlled trial in general practice. *Scand J Prim Health Care.* 15: 109-12, **1997**.

O'Sullivan DM & Tinanoff N. The association of early dental caries patterns with caries incidence in preschool children. *J Public Health Dent.* 56: 81-3, **1996**.

Peretz B, Ram D, Azo E & Efrat Y. Preschool caries as an indicator of future caries: a longitudinal study. *Pediatr Dent.* 25: 114-8, **2003**.

Petrolia DR & Bhattacharjee S. Revisiting Incentive Effects Evidence from a Random-Sample Mail Survey on Consumer Preferences for Fuel Ethanol. *Public Opinion Quarterly.* 73: 537-550, **2009**.

Poland C, 3rd & Hale KJ. Providing oral health to the little ones. *J Indiana Dent Assoc.* 82: 8-14, **2003**.

Pourat N, Andersen RM & Marcus M. Assessing the contribution of the dental care delivery system to oral health care disparities. *J Public Health Dent.* **2014**.

Ramos-Gomez F, UCLA Children's Dental Center Costs (Unpublished Data). In 2014.

Ramos-Gomez F, UCLA Dental IOCP Patient Retention Rates (Unpublished Data). In 2014.

Ramos-Gomez FJ. A model for community-based pediatric oral heath: implementation of an infant oral care program. *Int J Dent.* **2014**. PMC3920860.

Ramos-Gomez F, Chung LH, Gonzalez Beristain R, Santo W, Jue B, Weintraub J & Gansky S. Recruiting and retaining pregnant women from a community health center at the US-Mexico border for the Mothers and Youth Access clinical trial. *Clin Trials.* 5: 336-46, **2008**.

Ramos-Gomez FJ, Crall J, Gansky SA, Slayton RL & Featherstone JD. Caries risk assessment appropriate for the age 1 visit (infants and toddlers). *J Calif Dent Assoc.* 35: 687-702, **2007**.

Ramos-Gomez FJ, Huang GF, Masouredis CM & Braham RL. Prevalence and treatment costs of infant caries in Northern California. *ASDC J Dent Child.* 63: 108-12, **1996**.

Ramos-Gomez F & Ng MW. Into the future: keeping healthy teeth caries free: pediatric CAMBRA protocols. *J Calif Dent Assoc.* 39: 723-33, **2011**. PMC3457698.

Reisine S, Litt M & Tinanoff N. A biopsychosocial model to predict caries in preschool children. *Pediatr Dent.* 16: 413-8, **1994**.

Rogers T, Milkman KL & Volpp KG. Commitment devices: using initiatives to change behavior. *JAMA.* 311: 2065-6, **2014**.

Rozier RG, Sutton BK, Bawden JW, Haupt K, Slade GD & King RS. Prevention of early childhood caries in North Carolina medical practices: implications for research and practice. *J Dent Educ.* 67: 876-85, **2003**.

Ryu E, Couper MP & Marans RW. Survey incentives: Cash vs. in-kind; face-to-face vs. mail; Response rate vs. nonresponse error. *International Journal of Public Opinion Research.* 18: 89-106, **2006**.

Satterthwaite P. A randomised intervention study to examine the effect on immunisation coverage of making influenza vaccine available at no cost. *N Z Med J.* 110: 58-60, **1997**.

Savage MF, Lee JY, Kotch JB & Vann WF, Jr. Early preventive dental visits: effects on subsequent utilization and costs. *Pediatrics.* 114: e418-23, **2004**.

Seale NS & Casamassimo PS. Access to dental care for children in the United States: a survey of general practitioners. *J Am Dent Assoc.* 134: 1630-40, **2003**.

Sen AP, Sewell TB, Riley EB, Stearman B, Bellamy SL, Hu MF, Tao Y, Zhu J, Park JD, Loewenstein G, Asch DA & Volpp KG. Financial incentives for home-based health monitoring: a randomized controlled trial. *J Gen Intern Med.* 29: 770-7, **2014**. PMC4000326.

Shaffer VA & Arkes HR. Preference reversals in evaluations of cash versus non-cash incentives. *Journal of Economic Psychology.* 30: 859-872, **2009**.

Smith PB, Weinman ML, Johnson TC & Wait RB. Incentives and their influence on appointment compliance in a teenage family-planning clinic. *J Adolesc Health Care.* 11: 445-8, **1990**.

Stitzer ML, Rand CS, Bigelow GE & Mead AM. Contingent payment procedures for smoking reduction and cessation. *J Appl Behav Anal.* 19: 197-202, **1986**. PMC1308058.

Thornton RL. The Demand for, and Impact of, Learning HIV Status. *Am Econ Rev.* 98: 1829-1863, **2008**. PMC3115776.

Vargas CM, Crall JJ & Schneider DA. Sociodemographic distribution of pediatric dental caries: NHANES III, 1988-1994. *J Am Dent Assoc.* 129: 1229-38, **1998**.

Volpp KG, Pauly MV, Loewenstein G & Bangsberg D. P4P4P: an agenda for research on pay-for-performance for patients. *Health Aff (Millwood).* 28: 206-14, **2009**. PMC3507539.

Weintraub JA, Ramos-Gomez F, Jue B, Shain S, Hoover CI, Featherstone JD & Gansky SA. Fluoride varnish efficacy in preventing early childhood caries. *J Dent Res.* 85: 172-6, **2006**. PMC2257982.

Wing RR, Jeffery RW, Pronk N & Hellerstedt WL. Effects of a personal trainer and financial incentives on exercise adherence in overweight women in a behavioral weight loss program. *Obes Res.* 4: 457-62, **1996**.

SUPPLEMENTAL MATERIALS

These documents are relevant to the protocol, but they are not considered part of the protocol. They are stored and modified separately. As such, modifications to these documents do not require protocol amendments.

- Parent/Caregiver Informed Consent Form
- Questionnaire (baseline (first) visit)
- Questionnaire (2-month (final follow-up) visit)
- Oral Health Screening Form (with ADA Treatment Urgency Classification)
- Plaque Assessment Form
- Manual of Procedures

APPENDICES

Appendix A: Schedule of Events Table

APPENDIX A: SCHEDULE OF EVENTS

	Procedures	Screening/Baseline (Day 0)	Ongoing	Study Visit 2- Follow-up (Day 60 ± 28)	Ongoing (Delayed Control Group Only)	Study Visit 3- Follow-up (Day 120 ± 28)
	Signed Informed Consent Form	X				
	Assessment of Eligibility Criteria	X				
	Review of Medical/Dental History	X				
Questionnaires	Demographics, Acculturation, Health Literacy & Numeracy	X				
Questionnaires	Knowledge, Attitudes, Behaviors, Self-Efficacy	X		X		
Questionnaires	POQL	X		X		
Questionnaires	Other Family Member Behaviors	X		X		
Clinical Procedures	Dental Screening (Treatment Need / Urgency)	X		X		X
Clinical Procedures	Plaque Disclosing Solution Application & Ratings	X		X		
Clinical Procedures	Extraoral Photos	X		X		
Clinical Procedures	Toothbrush & Toothpaste Pump Demonstration (Prophylaxis)	X				
Clinical Procedures	Fluoride Varnish Application	X				
	Anticipatory Guidance	X		X		X
	Study Intervention		X	X		
	Toothbrushing Reports (smart powered toothbrush and parent/caregiver diaries)	X	X	X	X	X
	ChildPlus Dental Visiting Reports	X		X		
	SMS Text Messages	X	X	X	X	X
	Assessment of Adverse Events	(X)	(X)	(X)	(X)	(X)
