# Supplementary figures and images for: Monetary incentives for improving smartphone-measured oral hygiene behaviors in young children: A randomized pilot trial
Source: PLoS One. 2020 Jul 30;15(7):e0236692. doi: 10.1371/journal.pone.0236692 (PMC7392266; doi:10.1371/journal.pone.0236692)

S1 Figure. Trial accrual

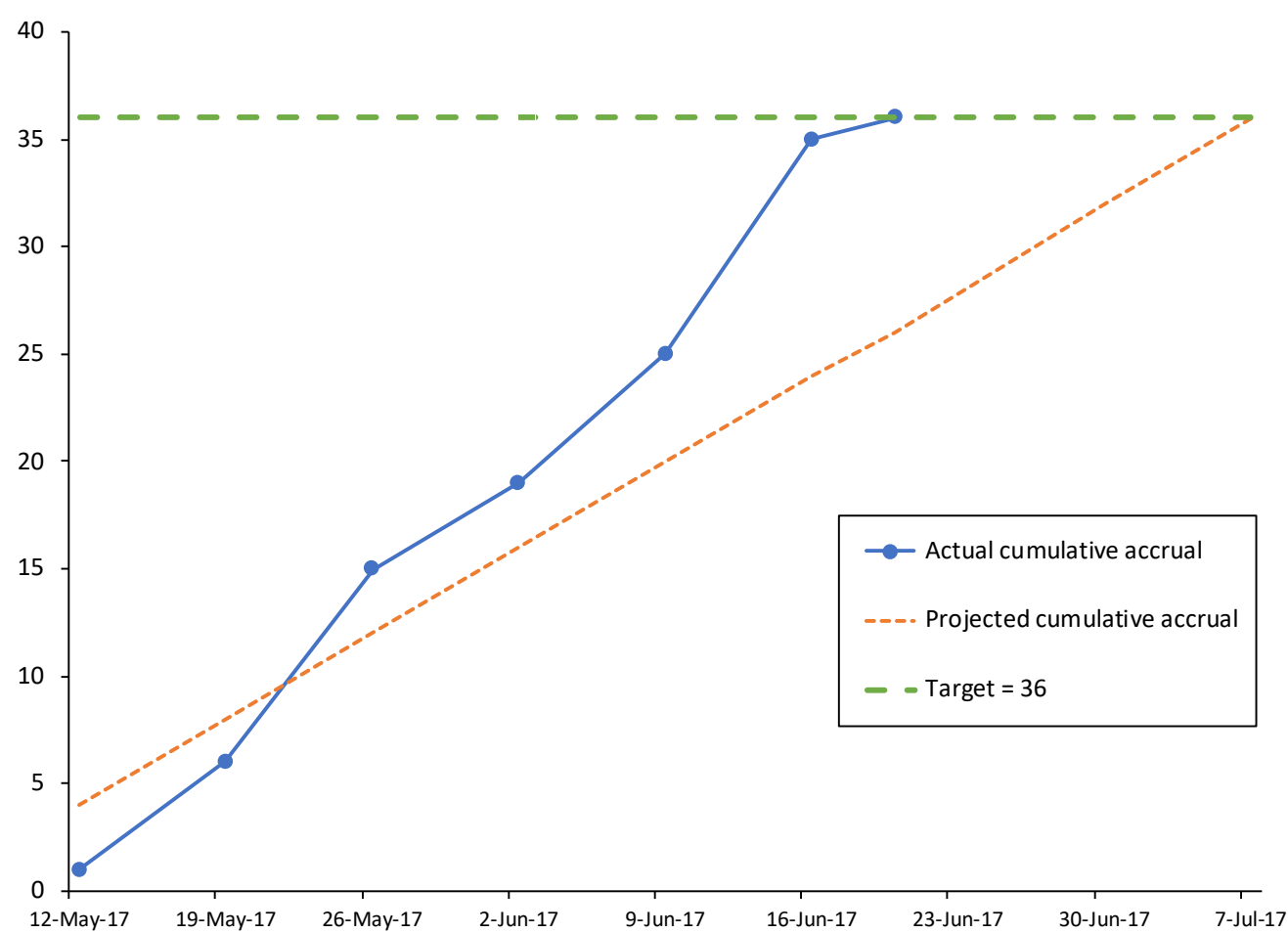

Supplement: S1 Fig — (PDF) [file pone.0236692.s003.pdf]

S5 Figure. In-person vs asynchronous plaque rating

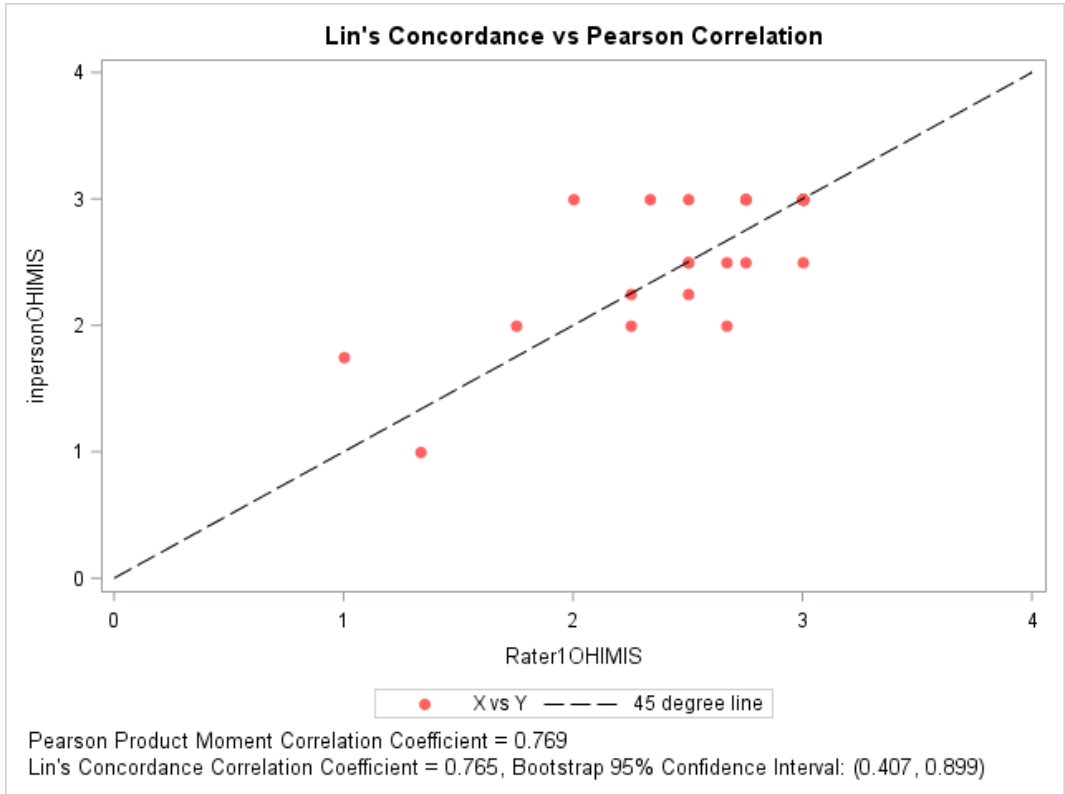

Supplement: S5 Fig — Pearson product moment correlation coefficient = 0.769. Lin’s Concordance Correlation Coefficient = 0.765, bootstrap 95% confidence interval (0.407, 0.899). (PDF) [file pone.0236692.s007.pdf]
