# Supplementary material for: Monetary incentives for improving smartphone-measured oral hygiene behaviors in young children: A randomized pilot trial
Source: PLoS One. 2020 Jul 30;15(7):e0236692. doi: 10.1371/journal.pone.0236692 (PMC7392266; doi:10.1371/journal.pone.0236692)
Supplement: S2 Fig — This figure shows a linear prediction of the fixed portion of the mixed-effects model with a random effect for child-parent dyad. Error bars represent 95% confidence intervals. (PDF) [file pone.0236692.s004.pdf]

S2 Figure. Effects of each incentive package on toothbrushing episodes by study week

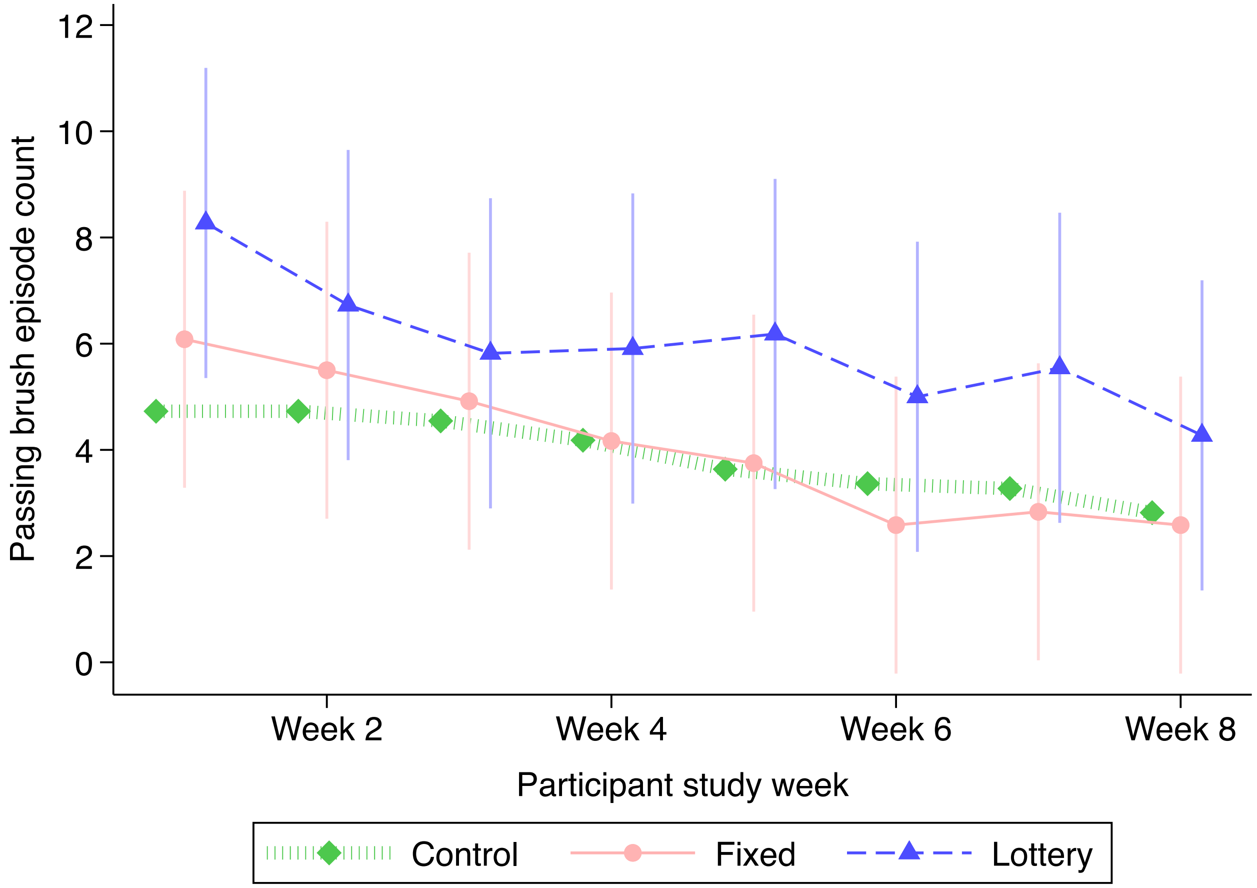

Note: This figure shows a linear prediction of the fixed portion of the mixed-effects model with a random effect for child-parent dyad. Error bars represent 95% confidence intervals.
