# Supplementary material for: Monetary incentives for improving smartphone-measured oral hygiene behaviors in young children: A randomized pilot trial
Source: PLoS One. 2020 Jul 30;15(7):e0236692. doi: 10.1371/journal.pone.0236692 (PMC7392266; doi:10.1371/journal.pone.0236692)
Supplement: S3 Fig — Linear mixed-effects model with a random effect for dyad. Purple text refers to contrasts of each incentive group against the control group. Error bars represent 95% confidence intervals. (PDF) [file pone.0236692.s005.pdf]

S3 Figure. Effects of each incentive package on toothbrushing episodes per week, by child age group

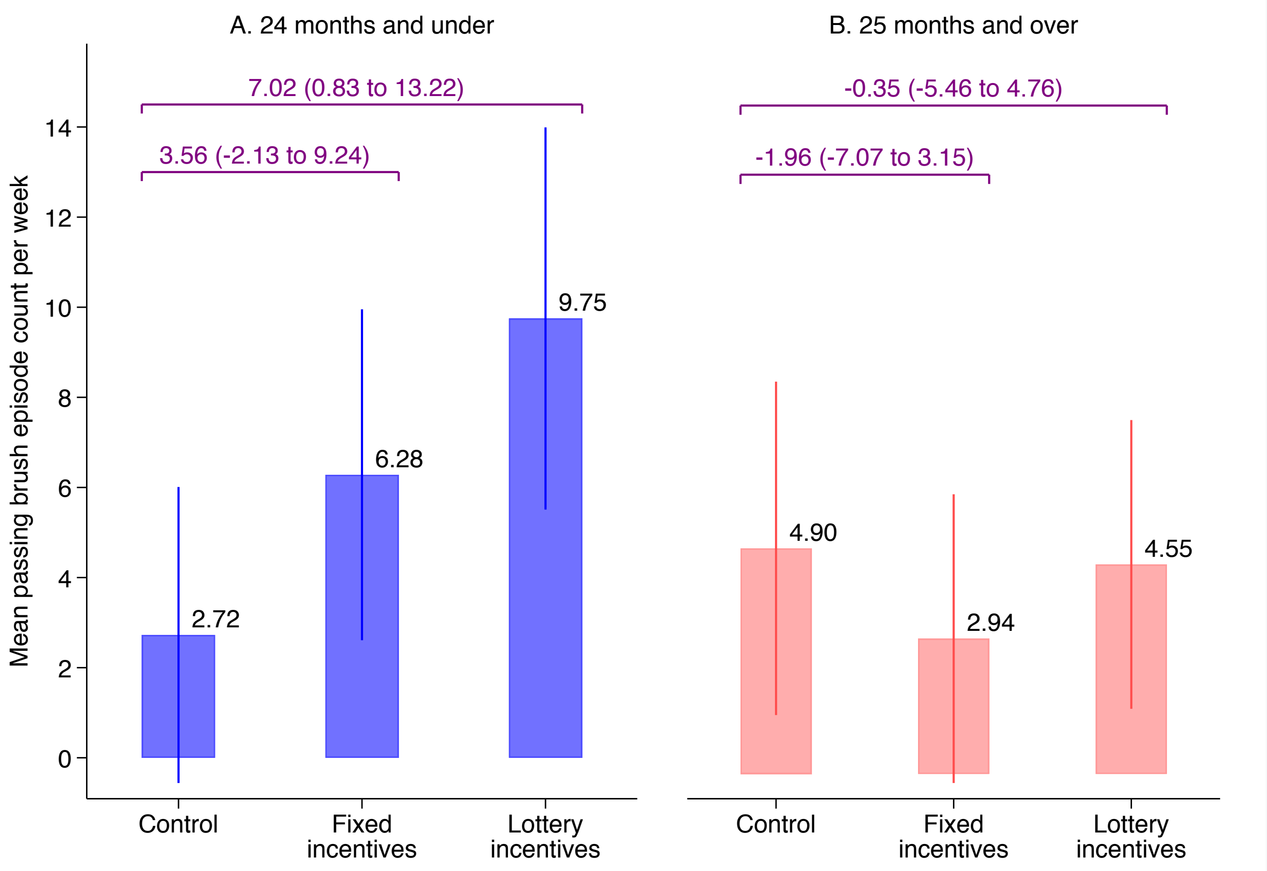

Note: Linear mixed-effects model with a random effect for dyad. Purple text refers to contrasts of each incentive group against the control group. Error bars represent 95% confidence intervals.
