# Supplementary material for: Monetary incentives for improving smartphone-measured oral hygiene behaviors in young children: A randomized pilot trial
Source: PLoS One. 2020 Jul 30;15(7):e0236692. doi: 10.1371/journal.pone.0236692 (PMC7392266; doi:10.1371/journal.pone.0236692)
Supplement: S4 Fig — The blue line denotes the regression line, AppCount = 4.36 + 0.33DiaryCount, R2 = 0.13. Markers are weighted by frequency of observations. (PDF) [file pone.0236692.s006.pdf]

S4 Figure. Association between parent-reported episodes per week (in diaries) and app-recorded episodes per week

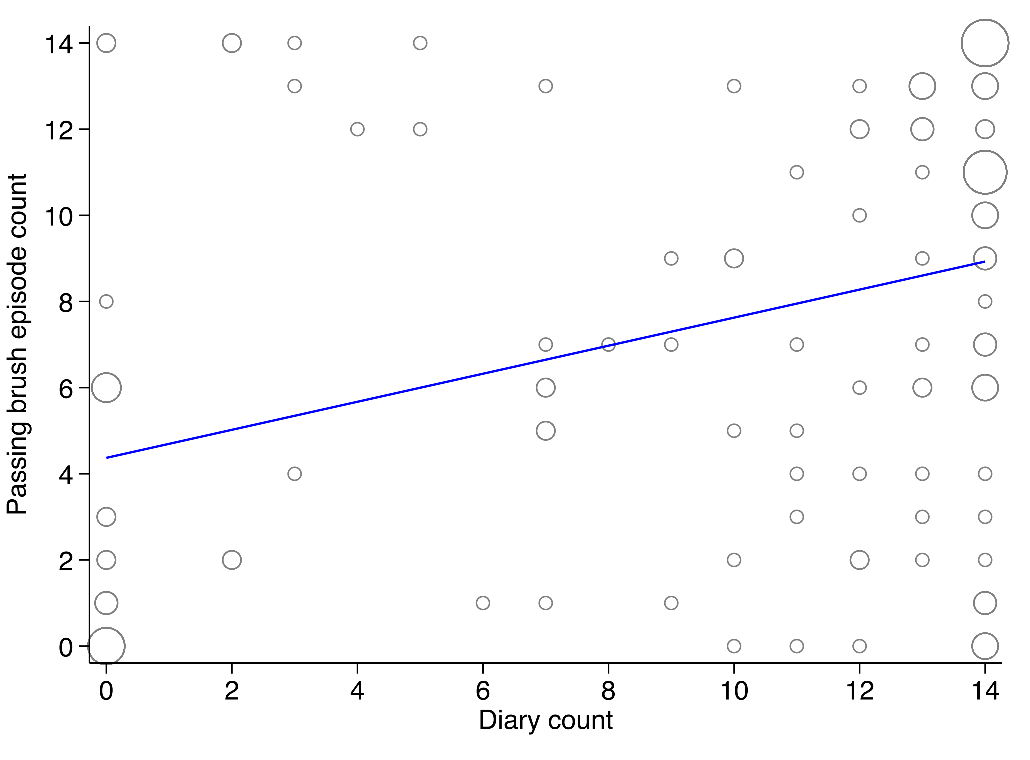

Note: The blue line denotes the regression line, $AppCount = 4.36 + 0.33DiaryCount, R^2 = 0.13$. Markers are weighted by frequency of observations.
