# Supplementary material for: Monetary incentives for improving smartphone-measured oral hygiene behaviors in young children: A randomized pilot trial
Source: PLoS One. 2020 Jul 30;15(7):e0236692. doi: 10.1371/journal.pone.0236692 (PMC7392266; doi:10.1371/journal.pone.0236692)
Supplement: S6 Fig — Mean cumulative earnings was $30.75 in the fixed incentives group (median $19.50) and $39.82 in the lottery incentives group (median $18.00). (PDF) [file pone.0236692.s008.pdf]

S6 Figure. Cumulative distribution of earnings by study group

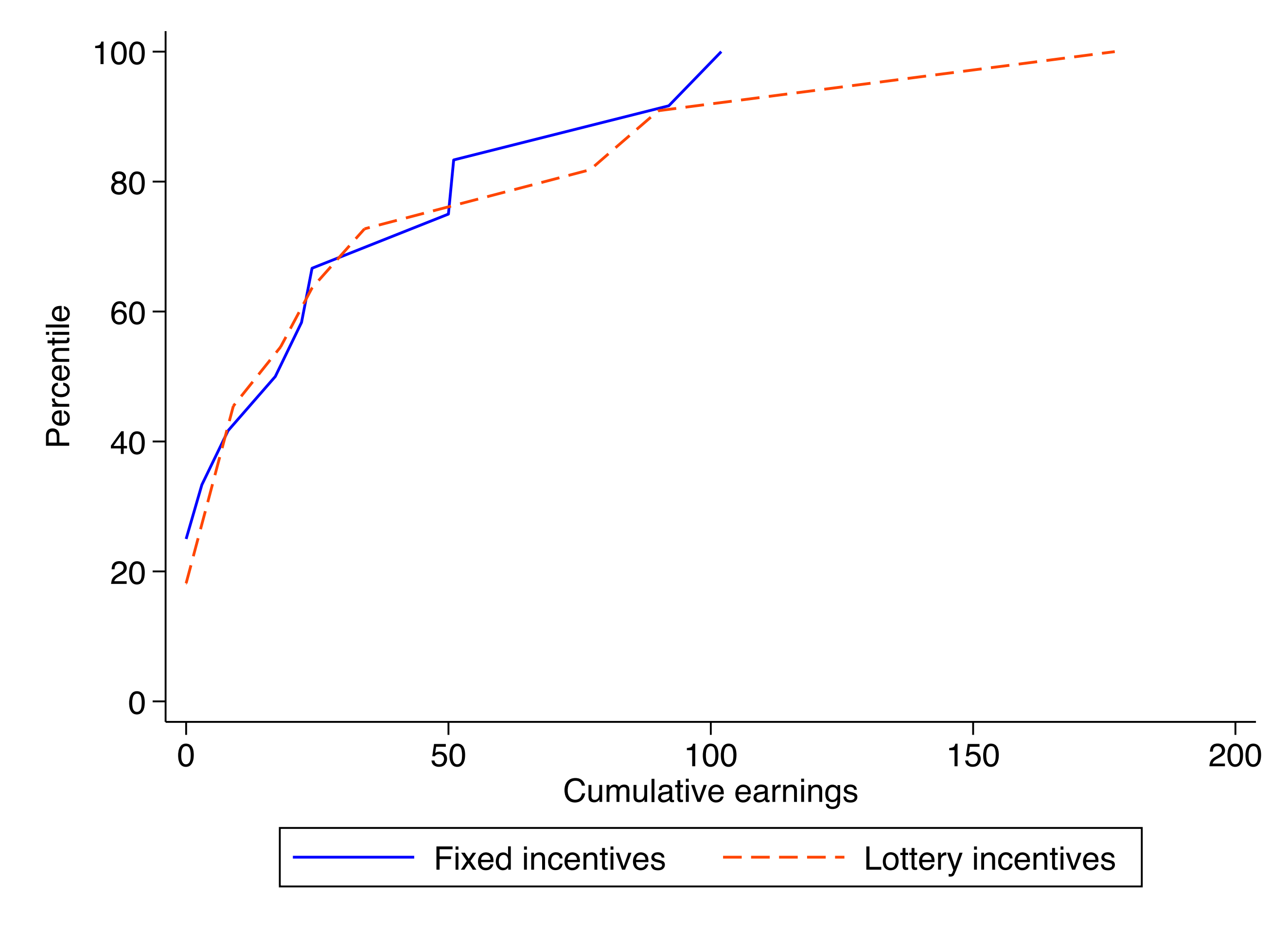

Note: Mean cumulative earnings was $30.75 in the fixed incentives group (median $19.50) and $39.82 in the lottery incentives group (median $18.00).
