# Supplementary material for: Monetary incentives for improving smartphone-measured oral hygiene behaviors in young children: A randomized pilot trial
Source: PLoS One. 2020 Jul 30;15(7):e0236692. doi: 10.1371/journal.pone.0236692 (PMC7392266; doi:10.1371/journal.pone.0236692)
Supplement: S1 Table — The low performance threshold was 7 qualifying episodes per week (roughly one per day). The high performance threshold was 14 qualifying episodes per day (twice per day). (DOCX) [file pone.0236692.s009.docx]

S1 Table. Details on lottery drawing using illustrative winning number of 38

| Lottery type | Rules | Prize | Chance of winning | Sample numbers that win each prize |
| --- | --- | --- | --- | --- |
| Low performance threshold | | |  |  |
| Small prize | Match 1 number in order | $25 | 18% | 32, 58 |
| Large prize | Match 2 numbers in order | $50 | 1% | 38 |
| High performance threshold | | |  |  |
| Small prize | Match 1 number regardless of order | $25 | 34% | 32, 23, 58, 85 |
| Large prize | Match 2 numbers regardless of order | $50 | 4% | 38, 83 |

Note: The low performance threshold was 7 qualifying episodes per week (roughly one per day). The high performance threshold was 14 qualifying episodes per day (twice per day).
