# Supplementary material for: Monetary incentives for improving smartphone-measured oral hygiene behaviors in young children: A randomized pilot trial
Source: PLoS One. 2020 Jul 30;15(7):e0236692. doi: 10.1371/journal.pone.0236692 (PMC7392266; doi:10.1371/journal.pone.0236692)
Supplement: S2 Table — In the message text, [X] denotes the number of brushing episodes that week; [Y] denotes the number of dollars earned for syncing that week ($0 or $3); [Z] denotes cumulative dollars earned to that point’ and [NM] denotes the two-digit winning lottery number that week. (DOCX) [file pone.0236692.s010.docx]

S2 Table. SMS message bank

| **Arm** | **Adherence threshold** | **Won small prize** | **Won large prize** | **Frequency** | **Message text** |
| --- | --- | --- | --- | --- | --- |
| **Reminder messages** | | | | | |
| All |  | . | . | Wed, Fri, Sun | BEECON: Please remember to sync your brush by the end of today. |
|  |  |  |  |  |  |
| **Feedback messages** | | | | | |
| Control | Below low-adherence  (< 1/day) | . | . | Tue after week’s end | BEECON: You brushed [X] times last week. You earned $[Y] for syncing. Total earned to date: $[Z]. |
|  | Met low-adherence (≥ 1/day) | . | . | Tue after week’s end | BEECON: You brushed [X] times last week. Nice job. You earned $[Y] for syncing. Total earned to date: $[Z]. |
|  | Met high-adherence (≥ 2/day) | . | . | Tue after week’s end | BEECON: You brushed [X] times last week. Great job! You earned $[Y] for syncing. Total earned to date: $[Z]. |
| Fixed incentive | Below low-adherence  (< 1/day) | . | . | Tue after week’s end | BEECON: You brushed [X] times last week. You earned $[Y] for syncing. Total earned to date: $[Z]. |
|  | Met low-adherence (≥ 1/day) | . | . | Tue after week’s end | BEECON: You brushed [X] times last week and earned $5. Nice job. You earned another $[Y] for syncing. Total earned to date: $[Z]. |
|  | Met high-adherence (≥ 2/day) | . | . | Tue after week’s end | BEECON: You brushed [X] times last week and earned $10. Great job! You earned another $[Y] for syncing. Total earned to date: $[Z]. |
| Lottery incentive | Below low-adherence (< 1/day) | No | No | Tue after week’s end | BEECON: You brushed [X] times last week. You did not win this week's drawing (winning # [NM]). You earned $[Y] for syncing. Total to date: $[Z]. |
|  | Met low-adherence (≥ 1/day) | No | No | Tue after week’s end | BEECON: You brushed [X] times last week. Nice job. You did not win this week's drawing (winning # [NM]). You earned $[Y] for syncing. Total to date: $[Z]. |
|  | Met high-adherence (≥ 2/day) | No | No | Tue after week’s end | BEECON: You brushed [X] times last week. Great job! You did not win this week's drawing (winning # [NM]). You earned $[Y] for syncing. Total to date: $[Z]. |
|  | Below low-adherence (< 1/day) | Yes | No | Tue after week’s end | BEECON: You brushed [X] times last week. You would have won $25 for brushing more (winning # [NM]). You earned $[Y] for syncing. Total to date: $[Z]. |
|  | Met low-adherence (≥ 1/day) | Yes | No | Tue after week’s end | BEECON: You brushed [X] times last week. Nice job. You won the $25 drawing for brushing (winning # [NM])! You earned $[Y] for syncing. Total to date: $[Z]. |
|  | Met high-adherence (≥ 2/day) | Yes | No | Tue after week’s end | BEECON: You brushed [X] times last week. Great job! You won the $25 drawing for brushing (winning # [NM])! You earned $[Y] for syncing. Total to date: $[Z]. |
|  | Below low-adherence (< 1/day) | No | Yes | Tue after week’s end | BEECON: You brushed [X] times last week. You would have won $50 for brushing more (winning # [NM]). You earned $[Y] for syncing. Total to date: $[Z]. |
|  | Met low-adherence (≥ 1/day) | No | Yes | Tue after week’s end | BEECON: You brushed [X] times last week. Nice job. You won the $50 drawing for brushing (winning # [NM])! You earned $[Y] for syncing. Total to date: $[Z]. |
|  | Met high-adherence (≥ 2/day) | No | Yes | Tue after week’s end | BEECON: You brushed [X] times last week. Great job! You won the $50 drawing for brushing (winning # [NM])! You earned $[Y] for syncing. Total to date: $[Z]. |
|  |  |  |  |  |  |
| **Lottery number selection** | | | | | |
| Lottery incentive | . | . | . | Sun | BEECON: Want to choose a number for this week's drawing? Reply with 1 number from 1 to 99. |

Note: In the message text, [X] denotes the number of brushing episodes that week; [Y] denotes the number of dollars earned for syncing that week ($0 or $3); [Z] denotes cumulative dollars earned to that point’ and [NM] denotes the two-digit winning lottery number that week.
