# Supplementary material for: Monetary incentives for improving smartphone-measured oral hygiene behaviors in young children: A randomized pilot trial
Source: PLoS One. 2020 Jul 30;15(7):e0236692. doi: 10.1371/journal.pone.0236692 (PMC7392266; doi:10.1371/journal.pone.0236692)
Supplement: S3 Table — Linear mixed-effects model with a random effect for child-parent dyad and 95% confidence intervals (in parentheses). Model 2 also includes indicators for study week. (DOCX) [file pone.0236692.s011.docx]

S3 Table. Effects of incentive packages on number of qualifying brushing episodes

|  | (1) | (2) |
| --- | --- | --- |
|  | Toothbrushing episodes | Toothbrushing episodes |
| Control (ref) |  |  |
| Fixed incentives | 0.14 | 0.14 |
|  | (-3.65, 3.94) | (-3.65, 3.94) |
| Lottery incentives | 2.06 | 2.06 |
|  | (-1.82, 5.93) | (-1.82, 5.93) |
| Constant | 3.91 | 5.64 |
|  | (1.17, 6.65) | (2.81, 8.46) |
| sd(Constant) | 4.37 | 4.39 |
|  | (3.38, 5.66) | (3.39, 5.67) |
| sd(Residual) | 2.46 | 2.25 |
|  | (2.24, 2.69) | (2.05, 2.46) |
| Week indicators | No | Yes |
| No. observations | 272 | 272 |
| No. clusters | 34 | 34 |
| Log likelihood | -684 | -655 |
| Mean dependent var. for control group | 3.91 | 3.91 |

Note: Linear mixed-effects model with a random effect for child-parent dyad and 95% confidence intervals (in parentheses). Model 2 also includes indicators for study week.
