# Supplementary material for: Monetary incentives for improving smartphone-measured oral hygiene behaviors in young children: A randomized pilot trial
Source: PLoS One. 2020 Jul 30;15(7):e0236692. doi: 10.1371/journal.pone.0236692 (PMC7392266; doi:10.1371/journal.pone.0236692)
Supplement: S4 Table — This table shows pairwise correlation coefficients between toothbrushing measures. Number of observations for each comparison is in brackets. (DOCX) [file pone.0236692.s012.docx]

S4 Table. Pairwise correlation between toothbrushing measures

|  | Mean Bluetooth episodes per week | Mean diary episodes per week | Plaque score at follow-up (in person) | Plaque score at follow-up (iPhone) | Change in pump weight |
| --- | --- | --- | --- | --- | --- |
| Mean Bluetooth episodes | 1.0000 |  |  |  |  |
| per week | [34] |  |  |  |  |
| Mean diary episodes per | 0.1828 | 1.0000 |  |  |  |
| week | [17] | [18] |  |  |  |
| Plaque score at follow-up | 0.0812 | 0.0960 | 1.0000 |  |  |
| (in person) | [24] | [17] | [26] |  |  |
| Plaque score at follow-up | 0.0095 | -0.2396 | 0.6839 | 1.0000 |  |
| (iPhone) | [23] | [16] | [25] | [25] |  |
| Change in pump weight | 0.1047 | -0.0736 | 0.1360 | 0.0579 | 1.0000 |
| (in ounces) | [24] | [18] | [25] | [24] | [26] |

Note: This table shows pairwise correlation coefficients between toothbrushing measures. Number of observations for each comparison is in brackets.
