# Supplementary material for: Monetary incentives for improving smartphone-measured oral hygiene behaviors in young children: A randomized pilot trial
Source: PLoS One. 2020 Jul 30;15(7):e0236692. doi: 10.1371/journal.pone.0236692 (PMC7392266; doi:10.1371/journal.pone.0236692)
Supplement: S5 Table — Data for complete cases are presented “n/N (%)”. (DOCX) [file pone.0236692.s013.docx]

S5 Table. Selected secondary outcome measures by age group

|  |  | Age group | |
| --- | --- | --- | --- |
|  | Total | ≤24 months | >24 months |
| Child comfortable with smart powered brush | 12/26 (46%) | 3/11 (27%) | 9/15 (60%) |
| Child cooperated for baseline disclosing gel application | 168/565 (30%) | 30/207 (14%) | 138/358 (39%) |
| Child cooperated for baseline plaque image (iPhone) | 212/565 (38%) | 44/207 (21%) | 168/358 (47%) |

Note: Data for complete cases are presented “n/N (%)”.
